# Impact of RNase E and RNase J on Global mRNA Metabolism in the Cyanobacterium *Synechocystis* PCC6803

**DOI:** 10.3389/fmicb.2020.01055

**Published:** 2020-06-03

**Authors:** Marina Cavaiuolo, Carine Chagneau, Soumaya Laalami, Harald Putzer

**Affiliations:** UMR 8261, CNRS, Institut de Biologie Physico-Chimique, Université de Paris, Paris, France

**Keywords:** RNase E, RNase J, *Synechocystis*, mRNA degradation, transcriptome, RNA-Seq

## Abstract

mRNA levels result from an equilibrium between transcription and degradation. Ribonucleases (RNases) facilitate the turnover of mRNA, which is an important way of controlling gene expression, allowing the cells to adjust transcript levels to a changing environment. In contrast to the heterotrophic model bacteria *Escherichia coli* and *Bacillus subtilis*, RNA decay has not been studied in detail in cyanobacteria. *Synechocystis* sp. PCC6803 encodes orthologs of both *E. coli* and *B. subtilis* RNases, including RNase E and RNase J, respectively. We show that *in vitro Sy* RNases E and J have an endonucleolytic cleavage specificity that is very similar between them and also compared to orthologous enzymes from *E. coli, B. subtilis*, and *Chlamydomonas.* Moreover, *Sy* RNase J displays a robust 5′-exoribonuclease activity similar to *B. subtilis* RNase J1, but unlike the evolutionarily related RNase J in chloroplasts. Both nucleases are essential and gene deletions could not be fully segregated in *Synechocystis*. We generated partially disrupted strains of *Sy* RNase E and J that were stable enough to allow for their growth and characterization. A transcriptome analysis of these strains partially depleted for RNases E and J, respectively, allowed to observe effects on specific transcripts. RNase E altered the expression of a larger number of chromosomal genes and antisense RNAs compared to RNase J, which rather affects endogenous plasmid encoded transcripts. Our results provide the first description of the main transcriptomic changes induced by the partial depletion of two essential ribonucleases in cyanobacteria.

## Introduction

Frequent environmental changes require bacteria to reprogram gene expression, often by adjusting mRNA amounts. Modulation of mRNA steady state levels is achieved at the transcriptional level by switching genes on and off and at the post-transcriptional level by removing mRNAs. Ribonucleases (RNases) are major players of such post-transcriptional regulation and they come in two flavors, endo- and exoribonucleases (Laalami et al., 2014; Mohanty and Kushner, 2016).

In bacteria, mRNA degradation generally follows an all-or-none pattern and therefore the only efficient way to regulate mRNA decay is to control the steps initiating mRNA degradation. Studies notably of the Gram-negative and Gram-positive model organisms *Escherichia coli* and *Bacillus subtilis* have revealed the principal enzymes involved in this process. RNases E, J, and Y are likely the key players that define bacterial mRNA decay strategies and all prokaryotes have at least one or any combination of these three ribonucleases (Laalami and Putzer, 2011). The characterization of these very disparate enzymes has revealed a surprisingly similar endonucleolytic cleavage specificity and suggests that the major mRNA decay pathways in bacteria initiate with an internal cleavage event. This can be summarized by “different enzymes-similar strategies” (Laalami et al., 2014). However, despite this convergent evolution toward a similar ribonucleolytic activity there is ample room for species specific strategies of mRNA metabolism, including the formation of degradosome complexes, subcellular compartmentalization and the presence of one or more of these key ribonucleases.

Cyanobacteria in general whose genomes have been sequenced have RNase E and RNase J, but no RNase Y orthologs. RNase E is an endonuclease present in many bacteria and plays an important role in all aspects of RNA metabolism. It forms large molecular degradosome complexes including among others a helicase and the 3′-5′ exoribonuclease polynucleotide phosphorylase (Mackie, 2013). Cyanobacterial RNase E has previously been shown to cleave *E. coli* 9S RNA and RNA I *in vitro* at the same sites as the *E. coli* enzyme (Kaberdin et al., 1998) and has been classified together with the *E. coli* orthologs from actinobacteria and proteobacteria as belonging to type I enzymes (Ait-Bara and Carpousis, 2015). However, while *E. coli* RNase E is anchored to the inner membrane through a membrane targeting sequence (MTS) (Strahl et al., 2015) cyanobacterial RNase E has no discernable MTS and the sequence motif recognized by polynucleotide phosphorylase is completely different (Zhang et al., 2014). In addition, in *Synechocystis* RNase E is part of a signal transduction pathway in which mRNA is destabilized in the dark (Horie et al., 2007).

RNase J was first identified and characterized in *B. subtilis* which has two paralogous enzymes, RNases J1 and J2 (Even et al., 2005). These dual-function RNases possess both 5′->3′ exonuclease and endonuclease activity (Even et al., 2005; Mathy et al., 2007). The two proteins can form a heterodimeric complex (Even et al., 2005; Mathy et al., 2010), but only RNase J1 appears to have a more global role in mRNA turnover and processing of 16S and 23S rRNAs (Mäder et al., 2008; Redko and Condon, 2010). Multiple RNase J paralogs only occur in firmicutes and cyanobacteria encode a single RNase J protein more or less equally similar in primary sequence to *B. subtilis* RNases J1 and J2 but contain a specific C-terminal extension (Even et al., 2005).

In addition to their frequent occurrence in prokaryotes, orthologs of RNase E and J are ubiquitous in photosynthetic eukaryotes, where they act in the chloroplast (Schein et al., 2008; Sharwood et al., 2011; Liponska et al., 2018). This organelle is the site of photosynthesis within plant and algae cells and derives from the endosymbiosis of a cyanobacterium into an ancestral eukaryotic cell. Indeed, all cyanobacteria whose genome has been sequenced so far have both RNase E and RNase J enzymes. However, while some work highlighted the importance of post-transcriptional regulation by RNase E for a few transcripts (Horie et al., 2007; Behler et al., 2018), not much is known about RNase J. The genome-wide function of these two ribonucleases in mediating post-transcriptional regulation remain largely unexplored and their characterization is pivotal to understand the degree of conservation or divergence of regulation of gene expression between the evolutionary related genomes of cyanobacteria and chloroplasts.

*Synechocystis* sp. PCC 6803, hereafter referred as *Synechocystis*, is a model organism for cyanobacteria. Its genome consists of a chromosome (3.5 megabases) present in multiple copies and several plasmids of different sizes. The polyploidy level is highly variable and is influenced by growth phase and external parameters (Zerulla et al., 2016). Here, we investigated for the first time the global role of RNase E and J in the RNA metabolism of *Synechocystis*. To find potential targets of RNase E and J we employed RNA sequencing and differential expression analysis of strains that are partially depleted for these RNases. We discovered that depletion for RNase E leads to an increase in abundance of mRNAs from chromosomal genes and antisense RNAs (asRNA), while the effects of RNase J depletion were milder and involved mostly expression of plasmid-encoded genes. The role of RNase E and J for the maturation or degradation of selected substrates was confirmed by Northern blotting. Our analyses revealed some overlapping effects in the respective RNase mutant strains including maturation of non-coding RNA and of polycistronic mRNA.

## Materials and Methods

### *Synechocystis* Strains, Culture Media, and Growth Conditions

The glucose tolerant *Synechocystis* PCC6803 strain was grown photoautotrophically in BG11 liquid medium supplemented with sodium bicarbonate in Erlenmeyer flasks on an orbital shaker at 150–170 rpm at 25°C under continuous illumination (30 μmol photons m^–2^ s^–1^ light intensity). Strains were maintained on BG11 media solidified with 1% (w/v) Bacto-Agar. For selection, routine maintenance of mutants and subsequent analysis, solid and/or liquid media was supplemented with the spectinomycin (Spc) at 50, 100, or 200 μg/ml. Cell density was estimated by measuring OD_730 nm_ with a spectrophotometer.

### Construction of Mutants and Segregation Analysis

Partial deletion mutants of *rne* and *rnj* in *Synechocystis* were generated by targeted gene deletion using homologous recombination. Gene constructs were generated by overlapping PCR (Primers listed in Supplementary Table S5). ∼700 bp regions upstream and downstream of the gene of interest were amplified, respectively, with primers HP1994/HP1995 and HP1998/HP1999 for the *rne* gene and with HP2000/HP2001 and HP2003/HP2004 for the *rnj* gene. The coding sequence of a Spc antibiotic resistance gene was amplified either with primers HP1996/HP1997 or HP2001/HP1997 that contained 5′ extensions with ∼15–20 bp homology with the adjacent 5′ and 3′ fragments, respectively. Assembled fragments were cloned into plasmid pJRD184 after digestion with NcoI and PstI restriction enzymes. A WT spc resistant control strain (WT-Spc) was constructed by inserting a Spc cassette containing a promoter and a terminator sequence (amplified with primers HP2088/HP2114) in a neutral site of the genome between the two bidirectional gene units *sll0452* and *slr0271*. For homologous recombination ∼700 bp regions upstream and downstream of the insertion site were amplified with primers HP2086/HP2087 and HP2090/HP2091, respectively. Assembled fragments were cloned in the pCR2-Blunt II TOPO vector (Thermofisher). The resulting plasmids were transformed by natural transformation as follows: 5 ml of an exponentially growing culture was centrifuged at room temperature at 9,000 × *g* for 5 min. The pellet was suspended in 2 mL of fresh BG11 medium to reach OD_730 nm_ = 2.5 and then mixed with 20–30 μg of recombinant plasmid DNA and incubated overnight under agitation at 25°C with 30 μmol photons m^–2^ s^–1^ light intensity. Cells were spread onto antibiotic-free BG11 medium for recovery before being transferred to plates containing 5 μg/ml of Spc. Colonies were isolated and passaged several times on solid media containing increasing concentrations of antibiotic (up to 200 μg/ml) to favor the segregation of the mutation. *rne* and *rnj* gene replacement was analyzed by PCR with primers HP1994/HP1999 and HP2000/HP2004, respectively, while integration of the resistance cassette was determined by PCR using primers HP2086/HP2091.

### Expression and Purification of *Sy* RNase E and *Sy* RNase J

To generate RNase E and J constructs for recombinant protein production, the coding sequences of the two genes was amplified by PCR with primers HP1975/HP1976 and HP1977/HP1978 (Supplementary Table S5), respectively, using *Synechocystis* genomic DNA as template. Purified PCR products were cloned into pTYB1 by restriction with NdeI and BglII for the overexpression of an in-frame C-terminal hexahistidine-tagged protein (RNase E) and N-terminal hexahistidine-tagged protein (RNase J). Recombinant vectors were named pI-SyRne and pI-SyRnj. 6×His-tagged versions of *Sy* RNase J and *Sy* RNase E proteins were overexpressed in *E. coli* Rosetta (DE3) and BL21 carrying the rare tRNA expressing plasmids Codon+, respectively. Typically, 1 liter of cultures were grown at 37°C in LB medium supplemented with kanamycin and chloramphenicol to OD_600 nm_ of 0.5 and recombinant protein expression was induced by the addition of 1 mM IPTG and continuing growth for 3 h at 37°C. Bacteria were harvested by centrifugation, washed in buffer A (20 mM HEPES pH 8, 500 mM NaCl, 10% glycerol) and resuspended in buffer A supplemented with DNase I (10 mg/ml) and protease inhibitors. Cells were disrupted by sonication (six times for 1 m, 30 s pause on ice) and clarified by centrifugation (7000 rpm, 30 min, 4°C). For *Sy* RNase J, the supernatant was loaded on a Ni–NTA agarose resin equilibrated in buffer A, and eluted with a linear gradient from 200 to 300 mM Imidazole in Buffer A. Fractions containing RNase were pooled and the eluted proteins were dialysed against buffer A. For purification of *Sy* RNase E, pellet was resuspended by gentle agitation in a buffer B (Urea 9M, NaCl 0.2 M, Tris–HCl 50 mM pH 8.3), and clarified by centrifugation (7000 rpm, 30 min, 20°C). The supernatant was affinity purified on a Ni–NTA agarose resin equilibrated in buffer B and eluted with a linear gradient from 200 to 300 mM imidazole in Buffer B. Fractions containing RNase E were pooled and the eluted proteins were dialysed against buffer A. Dialysed proteins were purified a second time on a Ni–NTA agarose resin equilibrated in buffer A, eluted with a linear gradient from 200 to 300 mM imidazole in Buffer A and dialysed against buffer A. Concentration of purified proteins was determined by measuring the absorbance at 280 nm. Purified proteins were analyzed by Western Blot. After separation on 10% SDS-polyacrylamide gels, proteins were transferred to a nitrocellulose membrane (Bio-Rad) by electroblotting. The membrane was blocked with 5% skimmed milk powder in 1× Phosphate Buffered Saline (PBS) buffer. To detect RNase J and RNase E-His tag proteins, the membrane was incubated with 6xHis specific antibodies overnight at 4°C. After washing, the alkaline phosphatase-conjugated anti-rabbit IgG (Dianova, Hamburg, Germany) was added, and the blot was developed with a CDP^∗^ detection system (Roche Diagnostics). The blot was scanned using a Storm 860 PhosphoImager (Molecular Dynamics Sunnyvale, California). Visualization of the bands was carried out with the ImageQuant software (Molecular Dynamics).

A 529 amino acid version of *E. coli* RNase E carrying a 10x His tag at the N terminus was purified as described above for *Sy* RNase J. A shortened version of *Chlamydomonas* RNase J was purified as described previously (Liponska et al., 2018).

### RNA *in vitro* Transcription and Labeling

*In vitro* transcription with T7 RNA polymerase was performed according to manufacturer instructions (Promega) using a PCR fragment as template. For RNA synthesis of the *psbA2* 132 nt long transcript, a PCR template was prepared using oligonucleotides HP2223 and HP2224 carrying a T7 promoter (Supplementary Table S5). RNA synthesis of the *yitJ* RNA we performed according to Shahbabian et al. (2009). The *psbA2* 35 nt transcript was kindly provided by W. Hess from Freiburg University. 5′-triphosphorylated transcripts were continuously labeled by addition of α-(32P) UTP in the *in vitro* transcription reaction, as described previously (Shahbabian et al., 2009). 5′-monophosphorylated transcripts were 5′-end labeled with γ-(^32^P) ATP using T4 polynucleotide kinase (New England Biolabs) according to manufacture instructions. *In vitro* synthesized transcripts were purified from unwanted products by elution from a denaturing 8% polyacrylamide gel. Probes for Northern hybridization were generated by *in vitro* transcription from PCR-amplified templates including 50 μ Ci ^32^P-UTP. The reactions were incubated at 37°C for 1 h, then transcripts were purified by gel filtration on Sephadex G-50 columns.

### RNase Cleavage Assays

Cleavage reactions were set up in a volume of 20 μl containing 20 mM Hepes-KOH pH 8.0, 20 mM or 8 mM MgCl_2_, 100 mM NaCl, 35 μM of 5′-triphosphorylated or 5′- monophosphorylated labeled RNA substrate and 1 μg of enzyme. Reactions were incubated for 15 min or 30 min at 30°C, and stopped by the addition of 7 μl of 3× gel loading buffer (87.5% formamide, 0.05% xylene cyanol, 0.05% bromophenol blue and 5 mM EDTA). Control reactions were performed by incubating the substrate with the reaction buffer alone. Samples were extracted with phenol/chloroform and reprecipitated prior to gel analysis to avoid retention of RNA by the ribonuclease. The samples were analyzed on 20, 8, and/or 5% denaturing polyacrylamide urea gels. Radioactive signals were visualized by a Typhoon FLA 9500 biomolecular imager (GE Healthcare). Images were treated with Image J software.

### RNA Isolation

Fifty ml of *Synechocystis* cultures were grown to OD_730 nm_ = 0.5. After centrifugation, cells were washed with 1 ml of TSE buffer (10 mM Tris–HCl, pH 8, 100 mM NaCl, 1 mM EDTA). The cell pellet was resuspended in 200 μl of ice cold TSE buffer and mixed with 250 μl of ice-cold glass beads (ODP Zirkonia 0.1 mm beads), 25 μl of chlorophorm/isoamylalcohol (24:1), 6.25 μl of 20% SDS and 150 μl of phenol (pH 5). After heating for 2 min at 95°C, cells were vortexed on a Disruptor for 10 min at 4°C. Phases were separated by centrifugation, and the aqueous phase was extracted with phenol/water, phenol pH8 and once more with phenol/chloroform/isoamyl alcohol (25:24:1). RNA was precipitated with ethanol-LiCl and resuspended in 50 μl water. Genomic DNA was digested by treatment with RQ1 DNase (Promega) prior to analysis. Concentration and quality of RNA samples were examined using an Agilent 2100 Bioanalyzer.

### Northern Blot

RNA blot analysis was carried out using 5 μg of total RNA separated either on a 1% formaldehyde-agarose or 10% acrylamide-urea gels (10% PAA, 0.5 g/ml urea, 1× Tris–borate) and electroblotted onto Hybond N+ membranes (GE Healthcare). RNA was cross-linked to the membrane at 120 mJ cm–2 for 1 min. The RNA sizes were estimated using the RiboRuler Low Range RNA Ladder (Thermo Fisher Scientific). Membranes were prehybridized for 2 h at 65°C with hybridization buffer ROTI in glass tubes under continuous rotation. RNA probes were denatured at 80°C for 3 min, kept on ice for 2 min and then added to the prehybridized membranes for hybridization at 68°C overnight. Membranes were washed at 68°C with washing solutions I (2× SSC and 0.1% SDS), II (1× SSC and 0.1% SDS) and III (0.5× SSC and 0.1% SDS) for 15 min each. The signals were detected with a GE Healthcare Typhoon FLA 9500 imaging system. Northern hybridization on CRISPR3 was performed as described previously (Behler et al., 2018) using a radioactively labeled RNA probe spanning CRISPR3 spacers 1–4. 16S or 5S rRNA (5S) was used as loading control.

### Transcriptome Analysis

For transcriptome analysis, RNA samples were treated with the Ribo-Zero Kit for Bacteria to remove ribosomal RNA (rRNAs). Multiplex RNA-Seq libraries were prepared with the Illumina TruSeq Stranded Total RNA Sample Preparation and sequenced (HiSeq2000). The genome sequence of *Synechocystis* PCC6803 and its annotation file were retrieved from the NCBI https://www.ncbi.nlm.nih.gov/genome/13549?genome_assembly_id=300974: NCBI accession numbers for chromosome: NC_00091-1.1/BA000022.2; plasmid pSYSA: NC_005230.1/AP0-04311.1; plasmid pSYSG: NC_005231.1/AP004312.1; plasmid pSYSM: NC_005229.1/AP004310.1; and plasmid pSYSX: NC_005232.1/AP006585.1. The NCBI annotation was complemented with the genomic sequence coordinates of the 5′- and 3′-UTR and of the *cis*-asRNA annotation deduced from Georg et al. (2009), Kopf et al. (2014), Pei et al. (2017). Single-end 100-nt reads were mapped to the genome using bwa (mem) (Li and Durbin, 2009). Analysis of the mappings used: (i) samtools for processing, sorting, selection of uniquely mapped reads (e.g., reads that map to exactly one location within the reference sequence) and indexing of the alignment files; (ii) BEDtools for computing genome-wide per-base coverage from each strand (bedtools genomecov function) and for counting the number of reads aligned to gene feature for each strand (bedtools multicov function); the IGV browser for visualizing the alignment files (Li and Durbin, 2009; Quinlan and Hall, 2010; Robinson et al., 2011). RNA-Seq coverage profile was plotted using gnuplot. To exclude the possibility that the phenotype observed in our mutants resulted from the integration of the Spc cassette elsewhere in the genome, we aligned all reads using as sequence reference the Spc coding sequence and applied soft-clipping in the alignment parameters of bwa (mem). Soft-clipped reads have only one portion of the read mapped to the reference sequence and the other portion that differs substantially from the reference sequence at that location. By applying soft-clipping in the mapping, the portions of the read that did not match well to the Spc coding sequence on either side of the read were retained in the alignment. We extracted the unmapped bases from these soft-clipped reads and searched the genome for their location to verify the soft-clipped bases matched to sequences flanking the targeted locus. Multi-dimensional scaling (MDS) plot was produced using the plotMDS function in the edgeR package (Robinson et al., 2010) on the top 500 differentially expressed genes using counts per million (CPM) normalized gene counts. CPM were calculated as the number of reads per each gene feature normalized to the effective library size of each sample. The effective library sizes were computed using the Trimmed Mean of M-values (TMM) scaling factor implemented in the edgeR package. A matrix of counts (y) with rows associated with unique feature identifiers and columns associated with the individual libraries was generated using the DGEList function. TMM normalization (y <- calcNormFactors (y, lib.size = lib.size, method = “TMM”) was computed by taking as library sizes the total number of mapped reads for each dataset. For differential gene expression, transcript features with CPM > 1 in at least three libraries were retained for the analysis. Variance calculation, common dispersion and tagwise variations were estimated using the quantile-adjusted conditional maximum likelihood (qCML) method implemented in edgeR. These were used for the exact Test function. Gene features were considered as significantly up- or down-regulated in the mutant vs. WT samples if the log_2_ fold-change (FC) ratio was ≥0.7 or ≤−0.7 and the *p*-value adjusted for multiple testing False Discovery Rate (FDR) calculated using the Benjamini-Hochberg (BH) method in edgeR was equal or lower than 5% (FDR ≤ 0.05). The MA plot was generated by using “plotSmear” from the edgeR package. Gene Ontologies terms were retrieved from the Alcodb cyano database^1^. Protein localization was predicted using the PSORTb Subcellular Localization Prediction Tool version 3 (Yu et al., 2010). Raw sequencing data are deposited at the NCBI Sequence Read Archive accession number (SRA) PRJNA611475.

### Sequence Alignment and 3D Structure Modeling

The multiple sequence and structure alignment program PROMALS3D was used to generate the protein alignments of *Sy* RNase E and J protein sequences against their orthologs from *B. subtilis, Chlamydomonas reinhardtii* and *E. coli* using default parameters. For molecular modeling, three-dimensional (3D) structures were generated with the SWISS-MODEL^2^, an automated protein structure homology-modeling server. Visualization and superposition of model and template structures was obtained using the molecular graphics, modeling and simulation features of YASARA program^3^.

## Results

### The RNase E and RNase J Enzymes of *Synechocystis* 6803

**FIGURE 1:**
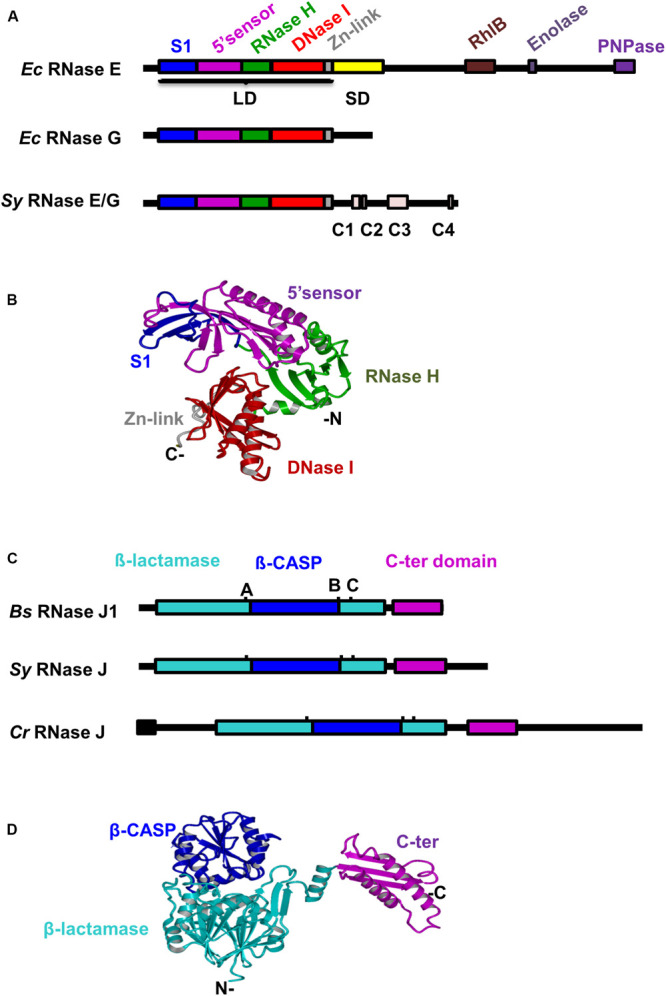
Conservation of domain structures of *Synechocystis* RNases E and J. **(A)** Alignment of RNase E/G proteins from *Synechocystis* and *Escherichia coli*. Conserved domains and motifs (Zhang et al., 2014) are depicted as differently colored filled boxes. LD, large domain; SD, small domain **(B)** The three-dimensional (3D) structure of *Sy* RNase E catalytic domain was generated with SWISS-MODEL using the *E. coli* RNase E crystal structure as template (PDB ID 2bx2, chain L). Relevant domains are indicated. **(C)** Alignment of RNase J proteins from *Synechocystis*, *Bacillus subtilis*, and *C. reinhardtii*. Conserved motifs of the β-lactamase superfamily **(A–C)** are indicated. The predicted chloroplast transit peptide is shown as black box. **(D)** The 3D structure of *Sy* RNase J was generated with SWISS-MODEL using the *T. thermophilus* RNase J crystal structure as template [PDB ID 3BK1, chain **(A)**]. Protein domains are shown in the same color code as in panel **(C)**.

A single gene product (*slr1129*, WP_010871286.1) with homology to *E. coli* (*Ec*) RNase E and RNase G is present in *Synechocystis* (*Sy*) (Figure 1A and Supplementary Figure S1A). The 674 amino acids (aa) long cyanobacterial protein *Sy* RNase E displays homology to the catalytic domains of *Ec* RNase E (36% identity/55% similarity) and *Ec* RNase G (37% identity/56% similarity). The catalytic domain of the *Sy* RNase E contains the four typical subdomains identified in the “large” domain of *Ec* RNase E (Callaghan et al., 2005) (aa 1–400: S1, 5′-sensor, RNase H and DNase I subdomains, Figure 1A), but it lacks the “small” domain classifying the protein as type IV RNase E (Ait-Bara and Carpousis, 2015). The C-terminal part of *Sy* RNase E does not share any sequence similarity with the *Ec* counterpart, but it contains four short sub-regions (named C1–C4) conserved in cyanobacterial RNase E proteins, among which C4 is critical for PNPase recognition (Zhang et al., 2014). Using the Swiss PDB Model, we performed a homology modeling of *Sy* RNase E based on the 3D structure of the *Ec* RNase E catalytic domain (Callaghan et al., 2005; Figure 1B). The best template was represented by the monomeric structure (PMD ID: 2bx2.1) of *Ec* RNase E. Besides the lack of the small domain, at the sequence level the predicted structure displayed 33.6% identity with that of its template and superposed with a root-mean-square distance (r.m.s.d.) of 0.45 Angstrom Å (Supplementary Figure S2).

RNase J orthologs are present in about half of the sequenced bacterial and archaeal genomes (Laalami et al., 2014). A BLAST search with the well-studied *B. subtilis* (*Bs*) RNases J1 and J2 sequences against the protein sequence database of *Synechocystis* identified a single gene product (slr0551, WP_010874094.1). With the exception of a specific 85 aa C-terminal extension the 640 aa long cyanobacterial protein displays high similarity with *Bs* RNases J1/2 enzymes over their entire length (Figure 1C and Supplementary Figure S1B). It shares 44% identical residues with RNase J1 (555 aa, 64% similarity) and 38% identical residues with RNase J2 (555 aa, 63% similarity). *Sy* RNase J also shares 35% identical residues with the central part of the 920 aa long *C. reinhardtii* (*Cr*) RNase J (aa 119–544; 55% similarity). Sequence analysis of *Sy* RNase J protein revealed the presence of all signature domains and motifs I-IV and A-C that assign this enzyme to the β-CASP family of metallo-β-lactamase ribonucleases (Supplementary Figure S1B). Because of the high conservation of the catalytic domain sequence between the RNase J proteins, we used the resolved 3D structure of the *Thermus thermophilus* RNase J (Li de la Sierra-Gallay et al., 2008) to construct a protein model for the cyanobacterial enzyme (Figure 1D). Except for an additional stretch of 10 aa in the C-terminal domain of *Sy* RNase J, the model and the template structures were superposed with a r.m.s.d. of 0.97 (Supplementary Figure S2).

### The Catalytic Activities of *Sy* RNases E and J

To characterize and compare the catalytic properties of *Sy* RNase E and J, recombinant His-tagged proteins were over-expressed in *E. coli* and purified (Supplementary Figure S3). When IPTG was added to cells containing plasmid pI-SyRne to induce *Sy* RNase E expression, we observed no obvious expression of a new protein with the expected molecular weight (MW), when cell lysates were examined on a SDS-polyacrylamide gel (Supplementary Figure S3A, lanes 2–4). Only a band corresponding to a MW of ∼110 kDa was observed in the insoluble fraction of the cell lysate (Supplementary Figure S3A, lane 2, depicted by an arrow). When this insoluble fraction was solubilized in urea and subsequently applied to a nickel-Sepharose column, a protein with an apparent MW of about 110 kDa was purified (Supplementary Figure S3B, lanes 2–5). Probing of this protein preparation with anti-His antibody (Supplementary Figure S3C) identified the same species of 110 kDa, indicating that this band corresponded to the full-length *Sy* RNase E. Although the predicted molecular mass of *Sy* RNase E is 78 kDa, the slower migration is possibly due to the presence of prolines (7.3%). This behavior is reminiscent of the similar aberrant migration of *Ec* RNase E (Casarégola et al., 1992; Kaberdin et al., 1998).

IPTG induction of *Sy* RNase J expression from the recombinant vector pI-SyRnj resulted in the overexpression of a protein with a MW of 75 kD (Supplementary Figure S3D, lanes 3–5), in accordance with its theoretical molecular mass. Probing of the *Sy* RNase J preparation from the soluble fraction with anti-His antibody identified the same band along with smaller products of ∼75 kDa (Supplementary Figure S3F, lane 1). These minor contaminating species may represent proteolytic products of full-length *Sy* RNase J.

**FIGURE 2:**
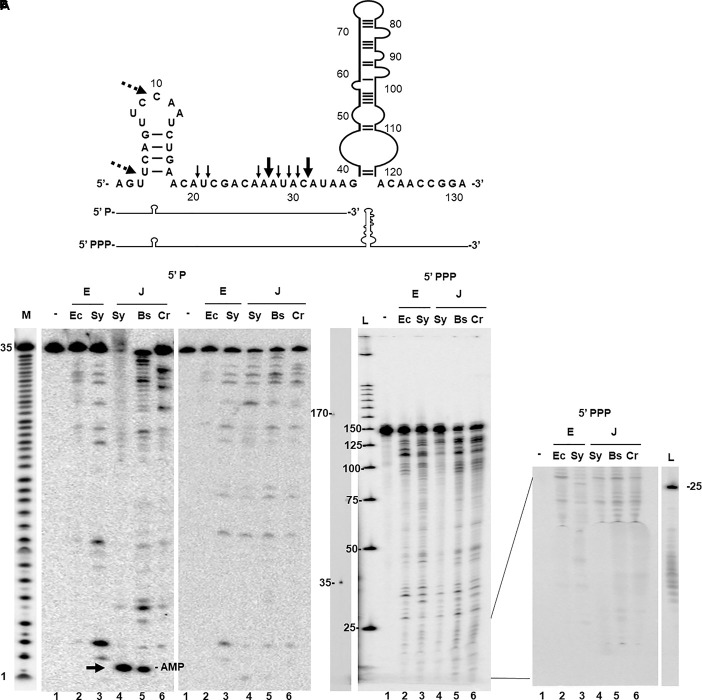
*In vitro* activity of *Sy* RNases E and J. **(A)** Sequence and secondary structure of a 132-nt *psbA2* transcript including the 5′-UTR (49-nt) and the first 80-nt of the coding sequence. Known *Sy* RNase E cleavage sites within 5′-UTR (Behler et al., 2018) are indicated by black arrows. Dashed arrows indicate additional cleavage sites by *Sy* RNase E identified in this study. The two transcripts (35 and 132 nt) used for the cleavage assays differ in their 3′ extension and the phosphorylation state of their 5′ end. **(B)** Cleavage assays were carried out on the 5′-monophosphorylated (P) – ^32^P end-labeled *psbA2* 5′-UTR (35 nt) using RNase E (E) from *Escherichia coli* (*Ec*) and *Synechocystis* (*Sy*) and with RNase J (J) from *Synechocystis* (*Sy*), *Bacillus subtilis* (*Bs*), and *C. reinhardtii* (*Cr*). As control (lane 1) the transcript was incubated with the reaction buffer only (-). Reactions were separated on a 20% urea PAGE gel directly (left panel) and after phenol extraction (right panel). M: alkaline hydrolysis ladder of the substrate. Mononucleotides liberated from the 5′-end are indicated by an arrow. They are lost after phenol extraction and precipitation. **(C)** Cleavage of the continuously labeled 5′-triphophorylated *psbA2* transcript (132 nt). Cleavage reactions were phenolized and separated on a 8% gel (left panel), and without phenolization separated on a 20% gel (only the bottom of the gel is shown, right panel). L = ^32^P-labeled 25 bp DNA ladder. Separately migrated RNA fragments of 175 and 35 nt are indicated.

We determined the cleavage activity of *Sy* RNase E and *Sy* RNase J on a known target of RNase E, the *psbA2* transcript (Horie et al., 2007; Behler et al., 2018). In *Synechocystis* the *psbA2* 5′-UTR harbors two major and six minor cleavage sites within an AU-rich sequence downstream of a short hairpin (Behler et al., 2018) as shown in Figure 2A. In order to obtain a meaningful comparison, the same substrate was also used to assay the activity of *Ec* RNase E (1–529 aa) a full length version of *Bs* RNase J1 (Even et al., 2005) and chloroplast *Cr* RNaseJ (1–590 aa) (Liponska et al., 2018). The enzymes were first tested on a synthetic 5′-monophosphorylated RNA corresponding to the first 35 nt of the *psbA2* 5′-UTR. Because the substrate was 5′ end-labeled, only the upstream fragment derived from cleavage could be detected (Figure 2B). The reactions were analyzed without (left panel) or after phenol extraction (right panel) because in the presence of *Sy* RNase J the RNA substrate was partially retained in the well (Figure 2B lane 4). *Synechocystis* and *E. coli* RNase E produced a similar endonucleolytic cleavage pattern differing essentially in the intensity of the 5′-cleavage products (Figure 2B). The general cleavage pattern of *Sy* RNase E was also in agreement with previous results (Behler et al., 2018). We also found two new additional sites (dashed arrows Figure 2A) that were not detected in Behler et al. (2018) probably because of difference of the staining and labeling methods.

*Synechocystis* RNase J as well as its orthologs from *B. subtilis* and *C. reinhardtii* have an almost identical endonucleolytical cleavage specificity which overall also strongly resembles that of the RNase E enzymes (Figure 2B). Furthermore, *Sy* RNase J also displayed 5′-exonucleolytic activity, producing mononucleotides, as the purified *Bs* RNase J1 (Figure 2B left panel, compare lanes 5 and 6). This was not the case for *Cr* RNase J for which no 5′-exonucleolytic activity was observed as expected (Liponska et al., 2018). Similar results were also observed on another transcript, the *yitJ* RNA (Supplementary Figure S4).

We also analyzed the sensitivity of *Sy* RNase J to the phosphorylation state of the mRNA 5′-end. To this aim, the enzymes were tested on a 5′-triphosphorylated (PPP) *psbA2* transcript of 132 nt identical in its 5′ sequence to the shorter 35 nt transcript described above but containing the entire 5′-UTR and the first 80-nt of the coding sequence (Figure 2A). The continuously labeled *in vitro* transcript allowed to detect both upstream and downstream fragments derived from cleavage. Again, all enzymes showed a similar endonucleolytic cleavage activity and specificity (Figure 2C, left panel). In addition, *Sy* RNase J just like Bs RNase J1 was not capable of exonucleolytically degrading the 5′ triphosphorylated transcript (Figure 2C) as no mononucleotides could be detected in significant amounts at the bottom of the gels (Figure 2C, right panel). We conclude that *Sy* RNase J has a dual activity: a 5′->3′ exoribonuclease toward 5′-monophosphorylated transcripts and an endoribonuclease activity with a cleavage specificity very similar to that of *Bs* RNase J1.

### Partial Depletion of *rne* or *rnj* Leads to Slow Growth and CRISPR3 RNA Maturation Defects

**FIGURE 3:**
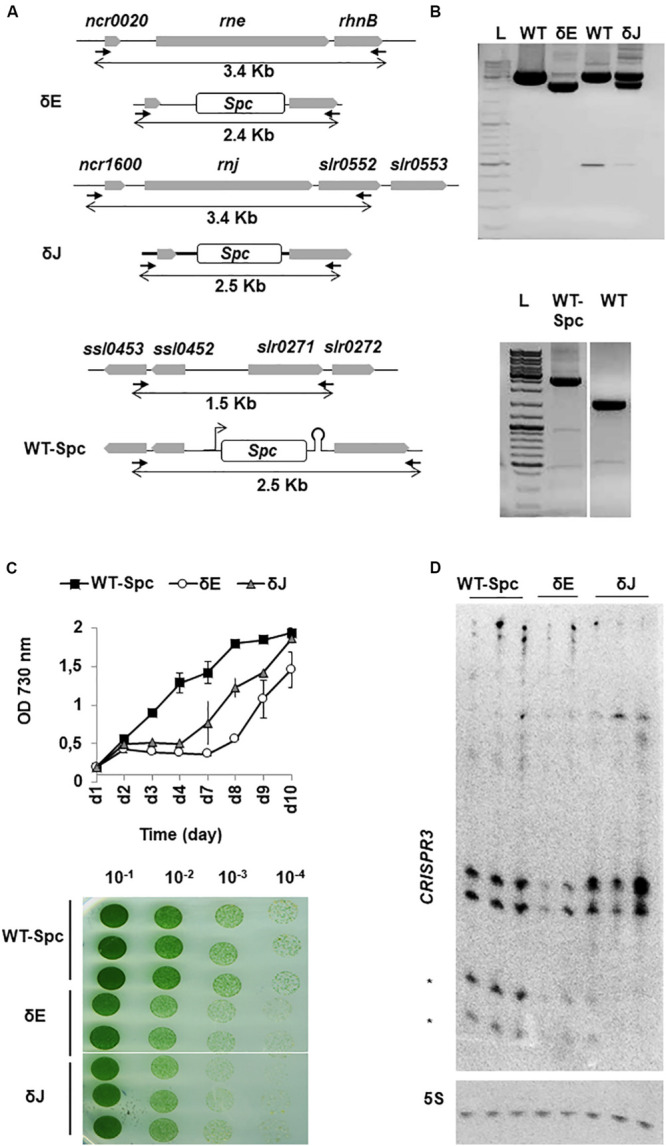
Deletion of *rne* and *rnj* genes in *Synechocystis*. **(A)** Schematic representation of mutant constructs. Genes are represented by gray boxes pointing in the direction of transcription. In δE and δJ strains the *rne* and *rnj* ORFs were replaced by a spc-resistance gene cassette (white box) by homologous recombination, while in the WT-Spc strain, the spc cassette was inserted between divergent transcriptional units. PCR primers used for segregation analysis are represented by black arrows; size of amplicons of WT and mutant chromosome loci are indicated by double arrows beneath each map. **(B)** PCR fragments of untransformed WT and mutant strains indicate the presence of the replaced (∼2.5 kb) and the WT (∼3.5 kb) chromosomal copies in δE and δJ strains (upper panel); the completely segregated insertion of the Spc cassette (∼2.5 kb) in the WT-Spc strain is shown in the lower panel. L = 1 kb DNA ladder. **(C)** WT-Spc, δE and δJ strains were grown in BG11 liquid and on solid medium supplemented with 200 μg/ml of Spc. Pre-cultures were diluted to OD_730 nm_ = 0.2. Growth was monitored for 10 days with mean values ± SD calculated from two independent experiments. Cells suspended to OD_730 nm_ = 0.2 were tenfold serially diluted in BG11 and spotted on solid medium. A representative of triplicate plates is shown. **(D)** Northern blot of CRISPR3 spacers 1–4. Mature crRNAs are indicated by asterisks. 5S rRNA (5S) was used as a loading control.

Attempts to completely delete the *rne* gene in cyanobacteria were not successful (Rott et al., 2003; Horie et al., 2007), strongly suggesting that RNase E is essential for cell viability. Whether RNase J is also required for cell growth is unknown. We thus attempted to delete both genes *rne* and *rnj* individually. The coding regions of the genes were replaced by a spectinomycin (Spc) resistance gene via homologous recombination (Figure 3A) and the segregation of the mutant genome copy was assessed by PCR using flanking primers (Figure 3B; details in Materials and Methods). We consistently failed to obtain a complete segregation of the disrupted genes, even after repeated rounds of sub-culturing on solid media with increasing Spc concentrations (up to 200 mg/ml spc). When re-plating at even higher antibiotic concentrations (Spc 500 μg/ml), the strains were no longer viable. We concluded that the expression of both RNase E and RNase J is required for cell viability in *Synechocystis*. However, the *rne* and *rnj* gene copies in the partially depleted strains was significantly reduced (Figure 3B) and we refer to these mutant strains as **δ**E and δJ, respectively. Since the mutant phenotype required the presence of spectinomycin we also constructed a reference control strain (WT-Spc), in which a Spc cassette was inserted into a neutral site of the genome between two divergent transcriptional units (Figure 3A). This insertion could be completely segregated as verified by PCR (Figure 3B, lower panel).

To determine the effect of each deletion on the physiology of *Synechocystis*, we compared the growth of each strain on solid medium and in liquid culture. Although not fully segregated, the δE and δJ strains exhibited retarded growth compared to the WT-Spc strain (Figure 3C). This effect was also clearly visible on solid medium supplemented with Spc (Figure 3C, lower panel). The WT-Spc grew, as measured by increase in OD_730 nm_, with an average doubling time of less than 24 h. Conversely, the δJ and δE strains exhibited an obvious decrease in the growth rate from the 2nd to the 4th or 7th day of growth, respectively, before entering in exponential phase, with the same doubling time as the WT-Spc. This delayed resumption of normal growth was likely due to a decreased effect of the antibiotic over time. In fact, the gene deletions were not stable in the absence of selective pressure from the added Spc : when grown without antibiotic, the mutants quickly reverted to WT phenotype, as indicated by growth rate and PCR analysis (Supplementary Figure S5).

We could not measure the remaining levels of ribonuclease in our mutants by Western blot. However, to assess that the phenotype observed in our mutants was indeed due to a reduction in the intracellular levels of the RNases, we searched for a defect in degradation/maturation of specific transcripts in the mutants compared to WT-Spc. CRISPR3 is a well-studied *in vivo* target of *Sy* RNase E (Behler et al., 2018). This long RNA is subjected to several cleavage events leading to two major species of small mature CRISPR RNAs (crRNAs) of ∼40 and 50-nt in length. For both mutants δE and δJ, total RNA was isolated from two or three cultures with OD_730 nm_ = 0.5, which derived from the same overnight pre-culture. Northern blot analysis of CRISPR3 revealed not only the previously observed decrease in crRNA levels in the δE mutants (Behler et al., 2018), but also in the δJ mutants (Figure 3D). While this result provided an *in vivo* evidence for a direct link between decreased crRNA processing and RNase E and J availability for CRISPR3 maturation (manuscript in preparation), it indicated that the partial gene deletions were sufficient to observe effects on degradation and/or maturation of RNAs.

### Transcriptomic Analysis of Partial *rne* and *rnj* Deletion Strains

Encouraged by the *in vivo* effects described above, we characterized the genome-wide effects of RNase E and RNase J depletion, respectively. To this aim, we sequenced the transcriptome of δE, δJ, and WT-Spc strains, using the same RNA preparations that had shown the effect on CRISPR3 maturation (Figure 3D). Using stranded RNA-Seq, a total of ∼372 million raw sequencing reads was obtained from the transcriptomic analysis of 8 samples, with an average per sample of ∼40-million reads (Supplementary Table S1) mapped to the *Synechocystis* genome. All samples had a mapping ratio of >70% and 0.2 – 0.9% of ribosomal RNA (rRNA)-based sequence reads, indicating a good overall sequencing accuracy and an efficient removal of rRNA, respectively.

To verify that the Spc cassette was inserted only at the intended locus in the genome, the RNA-Seq reads were also mapped to the Spc coding sequence alone using soft-clipping in the alignment parameters (see Materials and Methods). The unmapped portions on either side of the Spc soft-clipped reads matched only the 5′- (Supplementary Figure S6) and 3′-sequences flanking the targeted locus, that corresponded to 5′- and 3′-UTRs portions of the *rne* or *rnj* genes in the δE and δJ mutants, respectively. This analysis allowed us to conclude that the phenotype of our mutants was indeed due to the partial deletion of the *rne* and *rnj* genes, respectively, and not to the integration of the Spc cassette elsewhere in the genome.

**FIGURE 4:**
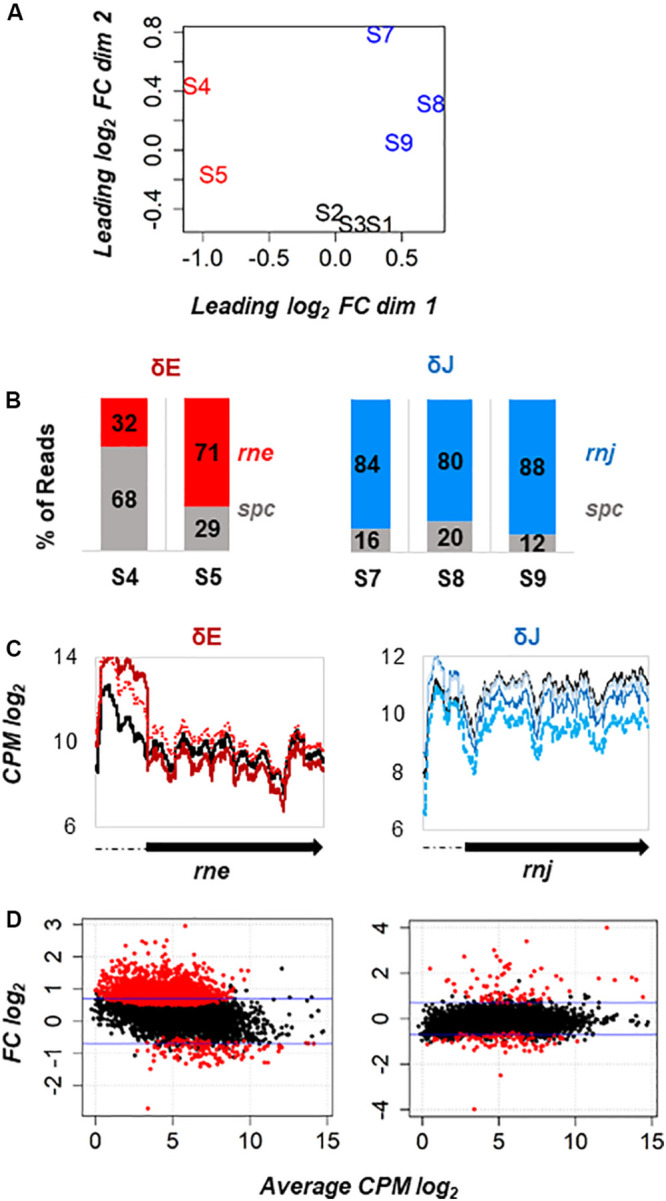
Transcriptome analysis of *rne* and *rnj* partial deletion strains. **(A)** Multidimensional scaling (MDS) plot of WT-Spc (S1, S2, S3), δE (S4, S5) and δJ (S7, S8, S9) libraries. Distance between samples is based on the leading log_2_ fold-change (FC) of the top 500 most differentially regulated genes. **(B)** Proportion of reads mapped to the Spc coding region (in gray) and to the *rne* (in red) and *rnj* (in blue) coding sequence with respect to the total spc+rne and spc+rnj reads, respectively, is shown for each mutant replicate. **(C)** Coverage profile expressed as log_2_ count per million (CPM) along the *rne* and *rnj* genes in each replicate: WT black solid line, S4 red solid line, S5 red dotted line, S7 blue dotted line, S8 blue solid line and S9 powder blue solid line. **(D)** MA plot of differentially expressed genes (red dots) at FDR < 0.05 identified in δE and δJ strains. The ordinate represents log2 FC.

To explore differential gene expression between WT-Spc, δE, and δJ strains, we measured the distance of gene expression values between any pair of samples. Our datasets included the analysis of ∼3610 open reading frames (ORFs) derived from the chromosome and the plasmids pSYS-M, pSYS-A, pSYS-G, and pSYS-X. In addition, 2785 previously compiled untranslated regions (UTRs, (Kopf et al., 2014) were also added to the analysis. On a multidimensional scaling (MDS) plot (Figure 4A), the WT-Spc samples (S1, S2, S3) clustered closely, indicating similar gene expression pattern and consistency between biological replicates. A certain degree of dissimilarity was instead observed among the δE (S4, S5) and δJ (S7, S8, S9) replicates. This was not unexpected since the essential nature of RNases E and J in polyploid *Synechocystis* likely leads to unpredictable numbers of WT and mutant copies in the different biological replicates of the mutants. Hence, to estimate the extent of such variability, for each replicate we calculated the proportion of reads coming either from the inserted Spc cassette and from the WT copy with respect to the total number spc+rne or spc+rnj reads, and we examined the coverage along the mutated locus (Figures 4B–C). The δJ replicates displayed from 12% to 22% of Spc reads, with a global decrease of the *rnj* transcripts ranging from 45- to 75-% of the WT levels (Figure 4C). On the other hand, the two δE replicates S4 and S5 displayed, respectively, ∼68 and ∼30% reads coming from the Spc cassette. Interestingly, reads coverage of the *rne* 5′-UTR increased in both replicates compared to WT (Figure 4C). Because the spc cassette was transcribed from the native *rne* promoter, this effect cannot be attributed to a higher transcriptional activity. We rather suggest that *Sy* RNase E cleave its own mRNA within the 5′-UTR, autoregulating its own expression in a similar way to what has been described for *E. coli* RNase E (Schuck et al., 2009). As a consequence, the different amount of reads along the *rne* coding sequence between the two replicates thus depends not only on the extent of gene deletion, but also on the competition of the endogenous *rne* and the chimeric *spc* transcripts for RNase E.

Figure 4D shows the MA plots of the expression data by taking into account the biological variability among the samples. When plotting the log_2_-FC (i.e., the log of the ratio of level counts for each gene between WT and mutant) against the log-average CPM (i.e., the average level counts for each gene across WT and mutant), most of the gene features had similar expression levels in WT and the mutant (black points around zero). However, at FDR ≤ 0.05, there were in total 2275 differentially expressed genes (DEGs) in the δE mutant. Among them, the expression levels of 769 ORF and 681 UTRs was more than 1.5-fold higher and those of 63 ORF and 51 UTR were more than 1.5-fold lower than the WT (Supplementary Table S2). Interestingly, the majority of DEGs had low counts. This suggests that weakly expressed genes might be preferentially targeted by RNase E in *Synechocystis*. Probably, their higher susceptibility to RNase E cleavage keeps their mRNA steady-state level lower. In the RNase J depleted strain, we observed 180 differentially expressed genes, among which the expression levels of 50 ORF and 29 UTRs were more than 1.5-fold higher and those of 50 ORF and 39 UTRs were more than 1.5-fold lower compared to WT (Supplementary Table S2).

### Functional Annotation of Differentially Regulated Transcripts in the δE and δJ Mutants

Differentially expressed genes (DEGs) with a log_2_-FC ≥0.7 and ≤−0.7 at a FDR ≤ 0.05 were categorized into functional groups according to Gene Ontology (GO) annotation and related cellular processes from the Alcodb cyano database (Table 1 and Supplementary Table S3). 40% of the GO annotated features (1502 in total) belonged to the functional categories “hypothetical” and “unknown,” but this is not surprising, since the function of nearly half of the annotated genes of *Synechocystis* is still not understood. In the δE mutant two functional categories were more enriched with DEGs: (1) genes involved in regulatory functions (∼7%), and (2) genes related to transport across membrane (∼8%). Nevertheless, other functional categories were equally represented in both mutants indicating that the two enzymes affect many different pathways in the cell but do not affect many transcripts of each pathway.

**TABLE 1:** Gene ontology classification from the Alcodb cyano database of 1502 differentially expressed transcript features in δE and δJ mutants.

**log_2_ ≤ −0.7**		**Process**	**log_2_ ≥ 0.7**	
**δJ**	**δE**		**δE**	**δJ**
2	4	Amino acid biosynthesis	42	0
0	4	Biosynthesis of cofactors, prosthetic groups, and carriers	79	4
0	0	Cell envelope	17	0
2	0	Cellular processes	23	2
1	0	Central intermediary metabolism	17	1
2	2	DNA replication, restriction, modification, recombination, and repair	30	0
5	5	Energy metabolism	29	0
1	1	Fatty acid, phospholipid and sterol metabolism	22	0
16	31	Hypothetical	377	29
5	2	Other categories	124	1
6	24	Photosynthesis and respiration	28	3
3	0	Pinnies, pyrimidines, nucleosides, and nucleotides	22	1
4	7	Regulatory functions	100	2
1	1	Transcription	9	0
0	5	Translation	55	2
10	13	Transport and binding proteins	121	2
22	20	Unknown	191	25

*For each GO category the number of up-regulated and down-regulated transcript feature (ORF, 5′-UTR and 3′-UTR) is indicated.*

In the δE mutant, most of the affected genes were located on the chromosome. The mRNA level of several important proteins was found to be dependent on the action of RNase E e.g., those involved in the initiation of chromosomal replication [DnaA (*sll0848*)], in cell division [FtsQ (*sll1632*)] and in translation [ribosomal protein S6 (*sll1767*)]. Interestingly, certain up-regulated genes in δE were orthologs of RNase E/G targets found in *Salmonella* (Chao et al., 2017) e.g., *slr1882* (riboflavin biosynthesis protein RibF), *slr1366* (lipoprotein signal peptidase) and *sll1632* (hypothetical protein) or in *E. coli* (Yajnik and Godson, 1993; Lee et al., 2002), e g., the *sll2012* (rpoD), sll0687 [RNA polymerase ECF-type (group 3) sigma factor]; other transcripts including e.g., *slr0446* (DNA polymerase III delta’ subunit dnaX), the 5′-UTR of *slr2023* ([acyl-carrier-protein] S-malonyltransferase FabD) and *sll0848* have been identified as targets of the functionally equivalent RNase Y protein in *Staphylococcus aureus* (Khemici et al., 2015) and in *B. subtilis* (Laalami et al., 2013).

Increased transcript levels were observed either for all the genes in an operon (e.g., *sll0634* and *sll0635* encoding, respectively, the photosystem I biogenesis protein BtpA and a probable thiamine-phosphate pyrophosphorylase), or for only some genes of an operon (e.g., *slr1045* and *slr1046* coding a hypothetical protein YCF63 and a putative TatA protein, respectively). Another striking example of a gene cluster regulated by RNase E was represented by the two adjacent genes *sll1392* and *slr1501* along with the downstream *slr1113*. Increased accumulation of these transcripts was observed in cells grown at pH 10 suggesting a role of this cluster in growth at high pH (Summerfield and Sherman, 2008). We also found other transcripts involved in resistance against reactive oxygen species (ROS) and response to ethanol stress, e.g., *slr1109* encoding an ankyrin homolog and *ssr2016* encoding the PGR5 protein (Yeremenko et al., 2005; Wang et al., 2012), in tRNA aminoacylation and modification, e.g., *sll1348* encoding epoxyqueuosine reductase, or in the glycolate pathway, e.g., *sll1349* coding a phosphoglycolate phosphatase.

Genes involved in multiple aspects of photosynthesis and respiration activities were also up-regulated. The expression level of *hliA* and *hliB* encoding high-light inducible proteins was higher in δE than WT. The two proteins are thought to maintain photosynthetic performance by absorbing excess excitation energy or enabling the cells to cope with elevated ROS (He et al., 2001). The mRNA encoding iron-stress chlorophyll-binding protein IsiA was also increased. The IsiA protein is notably important under iron-depletion conditions, where it forms an alternative antenna around photosystem I (Yeremenko et al., 2004).

Up-regulation of genes included in the transport and binding proteins category constituted a major response to the *rne* mutation (8%). Transcript abundance was increased for 73 chromosomal ORFs that encoded a diverse range of transport and binding proteins, including cation transporters and metal permeases. Among the up-regulated plasmid genes, we also found a cluster of genes, at coordinates 40–58 kb of the pSYS-M plasmid, involved in the biosynthesis and transport of polysaccharides. The cluster included four operon genes *sll5043*, *sll5044*, and *sll5046*, and two monocistronic genes *sll5052* and *sll5057*. Most of them encoded homologs of polysaccharide transporter and polysaccharide biosynthesis proteins as well as a sulfotransferase and glycosyl transferase.

RNase depletion influences transcript abundance by directly altering the stability and/or the proper maturation of an individual mRNA: if this mRNA encodes a protein regulator, for example, indirect effects affecting the expression of the associated regulon could be observed. Indeed, the δE mutant displayed increased levels of 100 features of transcripts encoding proteins with regulatory functions. In particular, expression of 10 response regulators (Galperin, 2006) among which the OmpR-type DNA-binding response regulator RpaA (*slr0115*), the NarL-type *sll1544*, *sll1708* and *sll1592*, the REC-HisK-type *slr1400*, unclassified *sll5059* and *sll5060*, the RsbU sigma factor SibG regulation protein *slr2031* and the *sll1423* gene coding the global transcription factor nitrogen control NtcA were increased. The latter was accompanied by variations in the expression of genes for the nitrogen assimilation pathway, which are all part of the NtcA regulon. These included for example urtA (*slr0447*), glnA (*slr1756*), Rre37 (*sll1330*), and gifB (*sll1515*) (Giner-Lamia et al., 2017).

In the δJ mutant 41 out of the 79 up-regulated gene features (Supplementary Tables S2, S3) were located on the pSYS-A, pSYS-X, and pSYS-M plasmids. Among them we found the *slr7095* gene involved in Toxin-Antitoxin systems for plasmid maintenance (Kopfmann and Hess, 2013) the *sll7078* transcript coding a potential regulator “TIGR03985,” a protein family member of CRISPR-associated (Cas) gene clusters, and the CRISPR2 precursor transcripts, both with possible functions in phage resistance (Scholz et al., 2013). The 38 remaining up-regulated gene features were on the chromosome and included those responding to different stresses such as low concentration of inorganic carbon (Ci) (*slr0374, slr0376, slr0373, slr1380, slr1379*) (Jiang et al., 2015) and cadmium resistance (*sll0381*) (Houot et al., 2007), suggesting a general stress response triggered after RNase J depletion. Interestingly, 35 up-regulated transcript features were observed in both the RNase E and RNase J depletion strains (Supplementary Table S2) among which 30 had GO annotation (Supplementary Table S2). Although we did not obtain a strong depletion phenotype, this may suggest that in *Synechocystis* there is no strategy to cope with the stress caused by the depletion of these RNases.

### The Effect of RNases E and J on Maturation and/or Abundance of Selected Substrates

**FIGURE 5:**
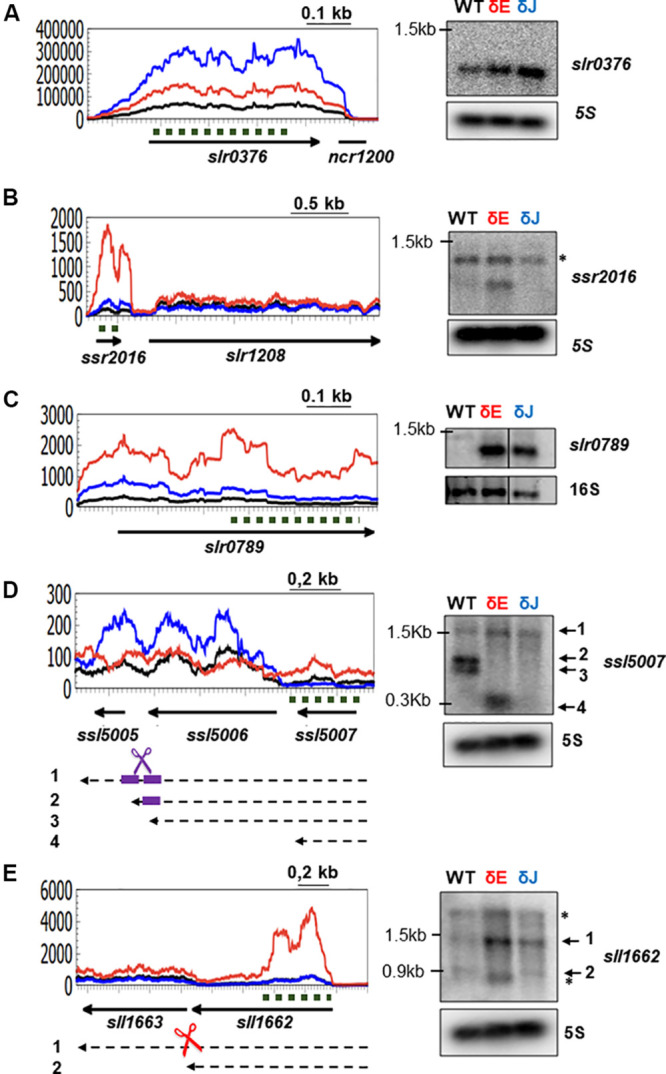
Analysis of specific transcript patterns in δE and δJ mutants. On the left, RNA-Seq coverage along the *slr0376* **(A)**, *ssr2016* **(B)**, *slr0789* **(C)**, *ssl5007* **(D)**, and *sll1662* **(E)** genes. The δE (in red) and δJ (in blue) strains are compared to the WT-Spc (in black). Arrows represent the ORF and the orientation on the genome; black dashed arrows represent the mRNA; dashed dark green lines indicate the probe used in the Northern blots (right panels). Scissors indicate an endoribonuclease cleavage: RNase E (red), RNase J (blue) and RNase E and J (violet). The two potential cleavage sites in **(D)** are indicated by thick violet short lines. 5S and 16S rRNA were used as loading control. Unspecific signals are indicated by asterisks.

To analyze RNase E and J related defects in RNA metabolism in more detail, we selected some of the genes that, based on the RNA-Seq data, showed enhanced transcript levels in the mutants. We focused on up-regulated genes since increased mRNA levels in the mutant is often an indication of the direct action of RNase E/J on the substrate RNAs. We performed Northern blot analyses on five different transcripts in order to determine mRNA levels as well as identify potential defects in mRNA processing patterns in the mutants compared to WT (Figure 5). All of the tested transcripts were in good agreement with RNA-Seq for consistency of the response, overall validating the accuracy of the differential expression analysis.

In *Synechocystis*, the *slr0376* gene encodes a hypothetical protein and it was reported to be transcribed either from its own promoter with the downstream non-coding RNA ncr1200 (Kopf et al., 2014) or as the last gene of an operon with the *slr0373* and *slr0374* (Jiang et al., 2015). *slr0376* is a stress responsive gene, since its transcript levels increased under low concentration of dissolved inorganic carbon (Ci) (Jiang et al., 2015). The read coverage profile (Figure 5A) indicated that *slr0376* is predominantly transcribed as a monocistronic mRNA. Compared to the WT-Spc strain, mutants depleted for RNase E or RNase J displayed an increased coverage over the *slr0376* gene. In agreement with RNA-Seq data, Northern blot analysis showed the presence of a single band corresponding in size to a monocistronic mRNA and its level increased in both mutants, more strongly in the RNase J depleted strain (Figure 5A).

The *ssr2016* gene is transcribed from its own promoter as a monocistronic mRNA (Kopf et al., 2014). It encodes a PGR5 protein required for FQR (ferredoxin: plastoquinone reductase) in cyclic electron flow around Photosystem I (Yeremenko et al., 2005). The gene is specifically induced by H_2_O_2_ and participates in ROS detoxification (Fedurayev et al., 2018). As judged from RNA-Seq coverage (Figure 5B), its mRNA levels were very low both in WT and δJ strains, while strongly increased in the δE mutants. Accordingly, a single band, corresponding in size to the *ssr2016* gene, could be only be detected in the δE mutant (Figure 5B).

The *slr0789* gene is the first gene of a bicistronic cluster. It encodes a protein belonging to the HPP family of integral membrane proteins, characterized by the presence of four putative transmembrane helices and a strongly conserved sequence motif His-Pro-Pro, with function in nitrite transport activity (Maeda et al., 2014). The level of this lowly abundant transcript was strongly increased in the two mutants as judged by RNA-Seq and Northern Blot (Figure 5C), representing a second example of a common target for RNase E and RNase J.

Our analyses uncovered also intriguing defects in mRNA processing in the δE and δJ mutants. In one set of affected genes, the accumulation of an unprocessed polycistronic precursor RNA occurred. The *ssl5007* gene encodes a protein of unknown function. RNA-Seq coverage indicated that it is a low abundant mRNA. When analyzed by Northern (Figure 5D), 3 bands were observed in the WT. A band of 1.5 kb probably corresponded to a polycistronic RNA encompassing the three genes *ssl5007*, *ssl5006*, and *ssl5005*. Two other transcripts were observed which according to the putative size encompasses the two genes in the cluster *ssl5007* and *ssl5006*. These two fragments are not present in the mutants suggesting a role for both RNase E and RNase J in a two sequential or simultaneous maturation of this precursor in a cooperative manner, potentially through an interaction of the two enzymes. A band corresponding in size to the *ssl5007* transcript was only detected in the δE mutant. This suggests that the *ssl5007* gene can be transcribed as monocistronic mRNA, which is a specific target of RNase E as deduced from RNA-Seq, its mRNA levels are strongly increased in the δE mutant.

A defect in processing was also observed for the *sll1662* gene, coding a prephenate dehydratase involved in amino acid and nitrogen metabolism. A 1.5 kb precursor RNA strongly accumulated in the δE mutant while the fragment of 0.9 kb that in size would correspond to a monocistronic *sll1662* mRNA was missing. Taken together, these examples suggest that RNase E and RNase J are involved in the processing of polycistronic precursor transcripts.

### Effects of RNase E and J Depletion on Antisense RNA Transcription

The genome of *Synechocystis* is pervasively transcribed with ∼64% of transcription start sites (TSSs) giving rise to antisense or small RNAs in a genome that is 87% protein coding (Georg et al., 2009). We therefore asked whether RNases E and J might be involved in the metabolism of these non-coding RNAs similar to what we have seen for mRNAs. However, in our RNA-Seq protocol small RNAs of ∼50–300-nt were excluded from the total RNA library preparation, limiting the analysis to RNAs of more than 300 nts. When we examined the RNA-seq coverage antisense to protein-coding genes (Supplementary Table S4A), we found that 415 and 58 gene features including ORF and UTRs had an increased expression (log_2_FC ≥ 0.7; FDR ≤ 0.05) in the δE and δJ mutants, respectively. In the δE mutant compared to WT, prominent examples included increased antisense coverage over *slr0604*, coding the lepA protein (Supplementary Figure S7), and *slr1494*, coding an ABC transporter. *cis* as-RNA species transcribed opposite to these genes were found by microarray (Georg et al., 2009) or evidenced from transcriptomic works (Kopf et al., 2014; Pei et al., 2017) (Supplementary Table S4B). Increased antisense coverage was also observed over genes for which no asRNA were identified or described in the literature suggesting that the repertoire of annotated *cis*-asRNAs is much larger.

## Discussion

In this work, we have investigated and compared the enzymatic activities of RNases E and J and studied their role in RNA metabolism in the cyanobacteria *Synechocystis.* A principal contribution of this study is the finding that *Sy* RNase J is a dual activity endo- and 5′ exoribonuclease, comparable to that of *B. subtilis* RNase J1 when assayed on the same 5′ monophosphorylated substrate. Moreover, *Sy* RNase J 5′ exonuclease activity was inhibited when the 5′-end of the substrate was tri-phosphorylated suggesting that the cyanobacterial enzyme (similar to *B. subtilis* RNase J1) cannot exonucleolytically degrade native transcripts in the cell. In addition, it appears that *Sy* RNase J (again similar to *B. subtilis* RNase J1) does not readily switch from endo- to 5′ exonucleolytic mode as the endonucleolytic cleavage products which are 5′ monophosphorylated remain stable during the *in vitro* reaction. One reason for this behavior might be that the structural context at the cleavage site (often close to a secondary structure) might not be favorable neither for switching nor for the binding of a new RNase molecule.

With respect to its activity *Sy* RNase J does not differ much from other characterized bacterial RNase J proteins. However, we have previously shown that the chloroplast targeted RNase J ortholog in *Chlamydomonas* has no significant 5′ exonuclease activity (Liponska et al., 2018). Chloroplasts are derived from a cyanobacterial ancestor and the major ribonucleases are clearly of prokaryotic origin (Stern et al., 2010). A comparison of the sequences of the chloroplast and cyanobacterial enzymes provides no clues as to why only the latter has a 5′ exonuclease activity and why the chloroplast enzyme might have lost the exo-activity despite its cyanobacterial origin.

The endonucleolytic activity of *Sy* RNase J assayed on a 5′ PPP cyanobacterial *psbA2* transcript was practically identical when compared to the cleavage patterns of both *B. subtilis* RNase J1 and RNase J of the *Chlamydomonas* chloroplast. Strikingly, the specificity of cleavage also resembled strongly that of *Synechocystis* and *E. coli* RNase E. All enzymes cleaved the *psbA2* leader at the same positions in a single-stranded region and in the loop of the stem-loop structure, with a bias toward A residues. The 5′ phosphorylation state of the transcript did not affect endonucleolytic cleavage efficiency, neither for the RNase J nor the RNase E proteins. However, more detailed comparative *in vitro* experiments are needed to make a meaningful conclusion on whether the activity of *Sy* RNase E can be affected by the 5′ phosphorylation state of the substrate as in *E. coli* (Jiang and Belasco, 2004; Jourdan and McDowall, 2008). Cyanobacteria diverged from the other bacterial species over 3 billion years ago. Despite this long evolutionary separation, the core domains of *Sy* RNase E and *Sy* RNase J retained very similar properties at the level of sequence, crucial active site residues and predicted 3D structure when compared with their orthologs in *E. coli* and *B. subtilis* (Figure 1 and Supplementary Figure S1). It is interesting that the cyanobacterial RNases E and J analyzed here show an almost identical endonuclease activity compared to their counterparts in Gram-negative and Gram-positive organisms and even in the chloroplast. This reinforces the concept that these completely unrelated ribonucleases have evolved through convergent evolutionary pressure toward an enzymatic activity that appears to be important for RNA metabolism across species boundaries.

Another important aspect of this work was to obtain a global view of the specific functions of RNase E and J in *Synechocystis* by identifying targeted transcripts *in vivo.* In the conditions used here, both RNase E and RNase J turned out to be essential. Indeed, we were unable to obtain fully segregated mutant strains where the respective genes were deleted on all chromosomes. The simultaneous presence of a wild type copy of the *rne* or *rnj* genes on some chromosomes together with gene deletions (spc cassette) present on other chromosomes made it difficult to obtain stable partially depleted strains. First, when removing the antibiotic pressure from the medium, the strains lost the Spc cassette quite rapidly. Second, especially for the *rne* deletion, the biological replicates displayed a different ratio of WT versus mutant chromosomal copies (spc mRNA, Figure 3), even when derived from the same pre-culture and grown under the same conditions. Such genetic instability has often been reported in cyanobacteria mutants (Angermayr et al., 2012; Du et al., 2017) and is still an issue that needs to be resolved. The instability of our partially segregated mutants is likely due to the essential nature of RNases E and J and may result from the random segregation of the WT and mutant chromosomal copies during cell division. Consequently, differences in the extent of segregation among the replicates contribute to variations in the number of RNA targets which are directly or indirectly affected by each mutation. This unavoidable heterogeneity of our mutant replicates therefore influenced the differential expression analysis of the RNA-Seq data. The partial depletion of *rne* and *rnj* gene copies, respectively, impaired photoautotrophic growth of *Synechocystis* in both solid and liquid media. This effect is most likely specific as the RNA-Seq analysis showed no significant effect on the transcript level of any other known ribonuclease in the mutants, with the exception of an increase of the RNA-DNA hybrid specific RNase HII (*slr1130*) in the *rne* mutant. However, since *rne* is co-transcribed with *slr1130* the increase in coverage observed over *slr1130* might simply be due to read-through transcription from the upstream spc cassette. Moreover, no cross-regulation of transcript levels occurred between RNase E and RNases J.

There is no obvious explanation for the growth phenotype of the RNase E and RNase J depleted mutants, notably for the delay observed in the mutants to enter exponential growth. Indeed, we observed changes in the expression of genes involved in many important physiological processes such as photosynthesis and respiration, transport, amino acid biosynthesis, and gene regulation (Table 1 and Supplementary Table S3). A common growth limitation for cyanobacteria is nitrogen shortage. That nitrogen assimilation could indeed be affected in the mutants was supported by the fact that genes coding proteins known to be activated during nitrogen starvation were altered in their expression. As an example, the NblA1 and NblA2 proteins are crucial for the degradation of phycobilisomes in the process of chlorosis after nitrogen starvation (Collier and Grossman, 1994; Baier et al., 2001) and their transcripts were down-regulated in the δJ mutant. NtcA is a regulator of genes associated with nitrogen metabolism. 51 genes are activated and 28 repressed directly (Giner-Lamia et al., 2017). The up-regulation of the gene coding the NtcA transcription factor in the RNase E mutant correlated with the increased or decreased levels of those genes that are differentially expressed after nitrogen starvation and that contain an NtcA binding site e.g., *slr2057*, *sll1665*, *slr0082*, and others mentioned in the results section. Overall, the pleiotropic effects of RNase E and RNase J depletion, respectively, on cyanobacterial physiology are compatible with their requirement for viability in *Synechocystis*. The different times needed to reach exponential growth might also reflect potentially different physiological states during which growth is observed.

For the transcriptome analysis of the RNase E and J depletion strains we used a 1.5-fold change in transcript levels compared to the WT as a threshold. Although it is possible that this cutoff may not be biologically relevant for all genes, we could validate several of the observed changes by Northern blot. On the other hand, using a rather stringent cut-off we might have missed some biologically relevant targets. Despite some difficulties to reliably reduce gene copy number, our study provides a first overview of the transcriptome-wide changes caused by reduced RNase E or RNase J expression. The effects observed here are not due neither to the use of the antibiotic in the media, since the WT control strain also contained a Spc cassette, neither to the integration of the cassette elsewhere in the genome (Supplementary Figure S6). The mRNA levels of RNase J were significantly reduced (Figure 4B and Supplementary Table S2) and likely correlate with a reduction in the intracellular levels of this RNase. For RNase E, the increased coverage over the 5′-UTR indicated higher stability of the mRNA, whose final levels also depended on the opposing effect caused by the gene deletion. In the case of RNase E it was possible to corroborate a predicted decrease in enzyme level through the direct observation of a reduced RNase E dependent maturation of CRISPR3 (Behler et al., 2018). By contrast, we observed no effect on the level of *psbA2* mRNA in all samples. This is not unexpected as our growth conditions under constant illumination, where *psbA* mRNA levels are very high, were very different from the reported dark-induced instability of *psbA2* mRNA which likely involves RNase E (Horie et al., 2007).

The quantity of DEGs detected in the mutants could also be affected by factors other than the extent of RNase depletion. First, RNA-Seq analysis was performed on RNA extracted from cells grown until the early exponential phase, when fewer genes are expected to be active or less abundant compared to late exponentially growing bacteria (Chang et al., 2002). Second, *Synechocystis* was grown in photoautotrophic conditions, but degradation, at least in the case of RNase E can be dark-induced for certain mRNAs (Horie et al., 2007). The largest functional gene category for both up- and down-regulated genes was hypothetical proteins/unknown function, comprising approximately 42% and 60% of the total quantity of differentially expressed genes in the *rne* and the *rnj* mutants, respectively. Nevertheless, RNA-Seq data suggested a global role of RNase E in the regulation of transcripts involved in different physiological, metabolic and cellular processes, including those that are essential to growth, e.g., DNA replication, mRNA translation and cell division.

Most often, cleavage by an endoribonuclease can be considered as the first step initiating the decay of an mRNA. Thus, transcripts that are up-regulated in an RNase mutant are more likely to represent direct targets. Depletion of RNase E led to a 1.5-fold increase in the steady-state level of ∼22% of the total non-rRNA-tRNA pool. Such an increase in mRNA levels was surprising considering the rather low level of depletion of RNase E and the variable extent of deletion among the mutant replicates. Interestingly, some of the up-regulated transcripts were orthologous of known RNase E targets in *Salmonella* or in *E. coli*, but this might not be significant given the large number of RNase E targets in these organisms.

Interestingly, 35% of candidate RNase E targets (up-regulated genes), for which we could predict a cellular localization (Supplementary Table S3), encoded proteins destined to the cytoplasmic membrane. In *E. coli*, RNase E is anchored to the inner membrane through a 15-aa long Membrane Targeting Sequence (MTS). While Cyanobacteria do not have a discernable MTS (Ait-Bara and Carpousis, 2015), the positively charged surface of its catalytic domain may contribute to membrane targeting (Murashko et al., 2012). Indeed, *Sy* RNase E protein used for the *in vitro* tests was purified from the insoluble fraction, while the soluble form was low abundant (data not shown). This may suggest that, as in *E. coli*, targeting of *Sy* RNase E to the membrane increases its activity and binding affinity to the RNA substrate (Murashko et al., 2012). The cellular localization of *Sy* RNase E could be due to a micro-domain e.g., one of the conserved sub-regions (Figure 1A; Zhang et al., 2014) C1 to C3 or to another interacting factor that targets the enzyme to a specific site in the cell.

One hundred and fourteen gene features (ORF and UTRs) showed reduced abundance and are more likely associated with changes in the steady-state levels of regulatory factors. Indeed, among the up-regulated genes in the *rne* mutant, we found transcriptional regulators, two-component signal transduction systems, and other regulatory players. Among the 146 genes annotated as putative transcriptional regulators (Kaneko et al., 2003), so far a dozen was functionally characterized to be implicated in the regulation of nitrite tolerance, iron limitation, acid tolerance and cadmium tolerance (Aichi et al., 2001; Michel and Pistorius, 2004; Ohta et al., 2005; Houot et al., 2007). Indeed, one example that illustrates well an indirect effect on reduced mRNA levels is the operon involved in the response to Cd. Expression of the repressor coded by the *slr1738* gene is increased in the δE mutant. All eight of the repressed targets genes identified in Houot et al. (2007) and corresponding to *slr0737*, *sll0427*, *slr1756*, *sll1270*, *slr1200*, *sll0108*, *sll1196*, and *slr0772* were down-regulated in our analysis (Supplementary Tables S2, S3).

A decrease in abundance of certain mRNAs could also be due to changes in the amount of non-coding RNAs, which are widespread in *Synechocystis* (Georg et al., 2009; Pei et al., 2017). Although for many of these non-coding RNAs the specific function is still unknown, they usually affect the stability/translation of their target mRNA through base-pairing. We found 415 cases (ORF and UTRs) where an antisense coverage increased upon depletion of RNase E (Supplementary Table S4), suggesting the involvement of asRNAs in the expression of the complementary mRNA. However, such increase was not always accompanied by a decrease in the level of its complementary mRNA. If asRNAs are dependent on RNase E for their maturation, in the absence of cleavage the asRNA may not be functionally active and the levels of the complementary mRNA are not affected. Alternatively, the abundance of the target mRNA is so low that even an increase in asRNA level would have little effect on its abundance. Another possibility is that asRNA can regulate translation of the complementary mRNA. The increased levels of asRNA over the *lepA* gene (Supplementary Figure S7) may induce an attenuation of translation by causing a pausing of the ribosome upstream of the double stranded RNA created by the asRNA: mRNA pairing. Indeed, the internal asRNA to lepA may affect protein biosynthesis as lepA encodes ribosomal back translocase, a protein only recently recognized as a third essential bacterial elongation factor (Georg et al., 2009).

In the RNase J depletion mutant, the number of up-regulated gene features (ORF and UTRs) was rather low, (59 in total), suggesting that RNase J although essential for growth only affects the levels of a small number of mRNAs. This contrasts to the situation observed in *B. subtilis* and *Helicobacter pylori*, where under more severe depletion conditions of RNase J up to 30% of mRNAs were affected (Mäder et al., 2008; Durand et al., 2012; Redko et al., 2016). However, at present we cannot conclude that in *Synechocystis* a smaller set of substrates is actually targeted by RNase J than by RNase E. Indeed, the real number of RNase J and RNase E targets are certainly underestimated and even at very low levels of RNase J the enzyme might still be sufficient to bind and cleave high affinity substrates. We noticed that, compared to the δE mutant, the transcriptomic changes were equally distributed between mRNAs whose abundance was increased and decreased in the δJ strain. At present, we don’t know whether an up-regulation of a potential regulator might be related to lower levels of certain mRNAs observed in the δJ mutant. Indeed, about half of the up-regulated genes encoded proteins whose function is not established.

Despite the few transcriptomic changes observed here in the δJ mutant, RNase J is required for cell survival in *Synechocystis*. No obvious candidate gene emerged from the list of transcripts whose abundance was specifically affected. As for RNase E, the majority of affected transcripts code either unknown or hypothetical proteins, the function of which might be important or essential for the bacterial physiology. The essentiality of RNase J could also simply be due to its contribution to the recycling of the nucleotide pool via its 5′ exonuclease activity that degrades RNA to mononucleotides.

RNA-Seq results pointed to a potential role of RNase J in the degradation or maturation of transcripts involved in defense (e.g., CRISPR-Cas adaptive immune system) and environmental stress responses. Most of the potential targets identified here are plasmid genes which may be responsible for functions important for the fitness of *Synechocystis*. Among them, the *slr5088*, encoded on pSYSM and annotated as probable short-chain dehydrogenases, was up-regulated. Increased levels of the Slr5088 gene product was found in engineered ethanol- (Pade et al., 2017) and lactic-acid-producing cells (Borirak et al., 2015). Although the function of this protein is not completely clear and might be involved in the turnover of lipids or organic acids, its expression is related to the stress imposed by lactic acid and ethanol production (Borirak et al., 2015).

Interestingly, depletion of RNase E and J, respectively, had a very similar growth phenotype. This may hint at a possible cooperation in the degradation/maturation of specific transcripts. Here, RNA-Seq revealed 35 transcripts features that were affected in both mutants. Common effects in the accumulation and processing of 4 transcripts was confirmed by Northern (Figures 3D, 5). These genes provide an example of common targets for both RNase E and RNase J and suggest that the two enzymes cooperate in degradation potentially by interacting between each other. However, whether RNase E and J might form a degradosome-like complex remains an open question.

Based on our *in vitro* assays, RNase E and RNase J can cleave endonucleolytically an RNA substrate at the same or similar positions. This implies that both enzymes might be able to compete for binding and cleavage of the same RNA substrate *in vivo*. Moreover, depletion of one RNase would then be at least partially compensated by the presence of the other RNase. However, for most transcripts it appeared that the critical functions of RNase E and J are non-overlapping in *Synechocystis*. RNase J displays a 5′->3′ exonuclease activity, at least *in vitro*. Therefore, the degradation of most transcripts would be more consistent with a model in which a first RNase E endonucleolytic cleavage is followed by RNase J 5′->3′ exonucleolytic trimming. If this is the case the number of targets determined by RNA-Seq represent an underestimation of the RNA substrates subjected to the combined action of RNase E and J. In fact, only if the endonucleolytic cleavage by RNase E occurs close to the 5′-end of a transcript and is followed by 5′->3′ degradation by RNase J, an increased RNA-Seq coverage over the whole length of the mRNA is observed in the *rnj* mutant. On the contrary, when RNAs is cleaved too close to the 3′-end, only a small fraction of the total length of the mRNA will be increased, resulting in no significant change in RNA-Seq coverage over the whole transcript in the *rnj* mutant. From the currently available data it is likely that *Sy* RNase E plays the major role in initiating mRNA decay through a first endonucleolytic cleavage, with RNase J degrading the downstream cleavage products exonucleolytically, much in the same way as RNase Y and RNase J1/J2 function in *B. subtilis.* However, the respective contributions of *Sy* RNases J and E to endonucleolytic cleavage *in vivo* remain to be analyzed, as well as the potential cooperative mode of function of the two enzymes in cyanobacterial mRNA metabolism.

## Data Availability Statement

The original contributions presented in the study are publicly available. This data can be found here: Link: https://www.ncbi.nlm.nih.gov/ Accession: PRJNA611475.

## Author Contributions

MC contributed to laboratory work, data analysis, and writing of the manuscript. CC contributed to laboratory work. HP contributed to writing and editing of the manuscript. SL and HP contributed to conceptualization, resources, and funding acquisition. All authors read and approved the manuscript.

## Conflict of Interest

The authors declare that the research was conducted in the absence of any commercial or financial relationships that could be construed as a potential conflict of interest.

## Footnotes

1 http://alcodb.jp/cyano/

2 http://swissmodel.expasy.org

3 http://www.yasara.org/

## Supplementary Material

FIGURE S1 Primary and secondary structure of *Sy* RNase E and *Sy* RNase J annotated using the PROMALS3D tool. **(A)** Multiple protein sequence alignment of RNase E/G proteins from *Synechocystis* and *E. coli* using the structure template of *Ec* RNase E (PDB ID: 2bx2, chain L). Conserved domains are underlined: RNase H in green, S1 in blue, 5′sensor in pink and DNase I in red; the C1 to C4 motifs are highlighted in cyano. α-helix predictions are colored in red and β-strand in blue. **(B)** Multiple protein sequences alignment of Sy RNase J, *Bs* RNase J1/J2 and *Cr* RNase J using the structure template of Tt RNaseJ1 (PDB ID: 3BK1 chain **(A)**. The β-lactamase motifs are in yellow, the β-CASP motifs in green. The β-CASP domain is underlined in blue and the C-terminal domain in pink. In **(A)** and **(B)** the consensus secondary structure (Consensus_ss) is indicated by h (α-helix) and e (β-strand). In the consensus amino acid sequence (Consensus_aa) conserved amino acids are in bold and uppercase letters; aliphatic: l; aromatic: @; hydrophobic: h; alcohol: o; polar residues: p; tiny: t; small: s; bulky residues: b; positively charged: +; negatively charged: −; charged: c.

FIGURE S2 Homology-based model of the *Sy* RNase and *Sy* RNase J structures. **(A)** The modeled catalytic region of *Sy* RNase E (blue) superimposed on the *E. coli* crystal structure PDB 2bx2, chain L (green). **(B)** The modeled structure of Sy RNase J (cyano) superimposed on the *T. thermophilus* crystal structure PDB 3BK1 (magenta).

FIGURE S3 Expression and purification of recombinant Sy RNase E **(A–C)** and Sy RNase J **(D–F)** in *E. coli*. SDS-PAGE was carried out on a 10% polyacrylamide gel and proteins were detected by Coomassie Brilliant Blue R-250 stain. **(A)** Induction of Sy RNase E: Lane 1, MW standards. Lanes 2 and 3, insoluble fractions after and before induction with 1 mM IPTG, respectively. Lanes 4 and 5, soluble fraction after and before induction, respectively. **(B)** Purification of *Sy* RNase J His-tagged recombinant protein. Lane 1, MW standards. Lanes 2 to 5, purified RNase E. **(C)** Immunoblot with anti-His tag antibody on collected pooled dialyzed proteins from lanes 3–5 of the gel in **(B)**. **(D)** Induction of *Sy* RNase J. Lane 1, MW standards; Lanes 2 and 3, whole cell lysates before and after induction, respectively. Lanes 4 and 5, insoluble fractions and soluble fraction after induction. **(E)** Purification of *Sy* RNase J His-tagged recombinant protein. Lane 1, MW standards. Lanes 2 to 8, purified RNase J. **(F)** Immunoblot with anti-His tag antibody. Pooled purified dialyzed proteins from lanes 1 to 4 (Lane 1) and from lanes 5 to 9, (Lane 2), respectively.

FIGURE S4 *In vitro* activity of *Sy* RNase E and *Sy* RNase J. A 5′-monophosphorilated (P) – 32P end-labeled yitJ was incubated with RNase E (E) from *E. coli* (*Ec*) and *Synechocystis* (*Sy*) and RNase J (J) from *Synechocystis* (*Sy*), *B. subtilis* (*Bs*), and *C. reinhardtii* (*Cr*). As control (lane 1) the transcript was incubated with the reaction buffer only (−). The reactions were separated on a 20% urea PAGE gel. The mononucleotides corresponding to liberated 5′-terminal nucleotide are at the bottom of the gel (depicted by an arrow) and are indicative of a 5′-3′ exoribonuclease activity. L = 32P-labeled 25 bp DNA ladder.

FIGURE S5 Genetic instability of *rne* and *rnj* deletions. **(A)** Growth curves of *Synechocystis* mutants and WT strains in the absence of antibiotic in BG11 medium. **(B)** PCR analysis of wild-type (WT-Spc) and mutant strains (δE and δJ) grown in the absence of spectinomycin. The almost exclusive presence of the WT gene copy (∼3.5 kb band) over the mutant gene copy (∼2.5 and ∼2.4 kb) in the mutant strains highlights the rapid loss of the partial gene deletions in the absence of antibiotic pressure.

FIGURE S6 Browser view of RNA-Seq soft-clipped reads showing the integration of the Spc cassette at the intended locus. Reads from replicates S4 **(A)** and S7 **(B)** are shown as representative examples. In these snapshots, the first 160-nt of Spc sequence is shown at the bottom. The soft-clipped portion at the 5′ end of the reads that do not match to the Spc coding sequence are shown as multicolor “rainbow” (C, blue; U, red; A, green; G, brown). The breakpoint of soft-clipped reads from reference is indicated by the arrow. The soft-clipped sequence corresponds to the sequence of 5′-UTR of *Sy* rne **(A)** and *Sy* rnj **(B)**.

FIGURE S7 Antisense coverage over lepA. RNA-Seq coverage over the lepA gene in the WT (black) and in the δE mutant (red), on the sense (+) strand (on the top) and on the antisense (−) strand (on the bottom). Arrow represents the lepA ORF and its orientation on the genome; the line represents the asRNA identified by Georg et al. (2009), RPM, reads per million.

TABLE S1 RNA-Seq mapping statistics.

TABLE S2 Differential gene expression analysis (sense coverage). List of up and down regulated protein coding genes (ORF and UTRs).

TABLE S3 Functional annotation from the Alcodb cyano database of differentially expressed protein coding genes (ORF and UTRs).

TABLE S4 Differential gene expression analysis (antisense coverage) and list of up and down features corresponding to previously annotated antisense RNA.

TABLE S5 Primers list.

## References

[B1] Aichi M. Takatani N. Omata T. (2001). Role of NtcB in activation of nitrate assimilation genes in the cyanobacterium *Synechocystis* sp. strain PCC 6803. *J. Bacteriol.* 183 5840–5847. 10.1128/jb.183.20.5840-5847.2001 11566981 PMC99660

[B2] Ait-Bara S. Carpousis A. J. (2015). RNA degradosomes in bacteria and chloroplasts: classification, distribution and evolution of RNase E homologs. *Mol. Microbiol.* 97 1021–1135. 10.1111/mmi.13095 26096689

[B3] Angermayr S. A. Paszota M. Hellingwerf K. J. (2012). Engineering a cyanobacterial cell factory for production of lactic acid. *Appl. Environ. Microbiol.* 78 7098–7106. 10.1128/aem.01587-12 22865063 PMC3457509

[B4] Baier K. Nicklisch S. Grundner C. Reinecke J. Lockau W. (2001). Expression of two nblA-homologous genes is required for phycobilisome degradation in nitrogen-starved *Synechocystis* sp. PCC6803. *FEMS Microbiol. Lett.* 195 35–39. 10.1111/j.1574-6968.2001.tb10494.x 11166992

[B5] Behler J. Sharma K. Reimann V. Wilde A. Urlaub H. Hess W. R. (2018). The host-encoded RNase E endonuclease as the crRNA maturation enzyme in a CRISPR-Cas subtype III-Bv system. *Nat. Microbiol.* 3 367–377. 10.1038/s41564-017-0103-5 29403013

[B6] Borirak O. De Koning L. J. Van Der Woude A. D. Hoefsloot H. C. Dekker H. L. Roseboom W. (2015). Quantitative proteomics analysis of an ethanol- and a lactate-producing mutant strain of *Synechocystis* sp. PCC6803. *Biotechnol. Biofuels* 8:111. 10.1186/s13068-015-0294-z PMC4526308 26246854

[B7] Callaghan A. J. Marcaida M. J. Stead J. A. Mcdowall K. J. Scott W. G. Luisi B. F. (2005). Structure of *Escherichia coli* RNase E catalytic domain and implications for RNA turnover. *Nature* 437 1187–1191. 10.1038/nature04084 16237448

[B8] Casarégola S. Jacq A. Laoudj D. Mcgurk G. Margarson S. Tempête M. (1992). Cloning and analysis of the entire *Escherichia coli* ams gene: ams is identical to hmp1 and encodes a 114 kDa protein that migrates as a 180 kDa protein. *J. Mol. Biol.* 228 30–40. 1447789 10.1016/0022-2836(92)90489-7

[B9] Chang D. E. Smalley D. J. Conway T. (2002). Gene expression profiling of *Escherichia coli* growth transitions: an expanded stringent response model. *Mol. Microbiol.* 45 289–306. 10.1046/j.1365-2958.2002.03001.x 12123445

[B10] Chao Y. Li L. Girodat D. Forstner K. U. Said N. Corcoran C. (2017). *In vivo* cleavage map illuminates the central role of RNase E in coding and non-coding RNA pathways. *Mol. Cell.* 65 39–51. 10.1016/j.molcel.2016.11.002 28061332 PMC5222698

[B11] Collier J. L. Grossman A. R. (1994). A small polypeptide triggers complete degradation of light-harvesting phycobiliproteins in nutrient-deprived cyanobacteria. *EMBO J.* 13 1039–1047. 10.1002/j.1460-2075.1994.tb06352.x 8131738 PMC394911

[B12] Du W. Angermayr S. A. Jongbloets J. A. Molenaar D. Bachmann H. Hellingwerf K. J. (2017). Nonhierarchical flux regulation exposes the fitness burden associated with lactate production in *Synechocystis* sp. PCC6803. *ACS Synth. Biol.* 6 395–401. 10.1021/acssynbio.6b00235 27936615

[B13] Durand S. Gilet L. Bessieres P. Nicolas P. Condon C. (2012). Three essential ribonucleases-RNase Y, J1, and III-control the abundance of a majority of Bacillus subtilis mRNAs. *PLoS Genet.* 8:e1002520. 10.1371/journal.pgen.1002520 22412379 PMC3297567

[B14] Even S. Pellegrini O. Zig L. Labas V. Vinh J. Brechemmier-Baey D. (2005). Ribonucleases J1 and J2: two novel endoribonucleases in *B. subtilis* with functional homology to E. coli RNase E. *Nucleic Acids Res.* 33 2141–2152. 10.1093/nar/gki505 15831787 PMC1079966

[B15] Fedurayev P. V. Mironov K. S. Gabrielyan D. A. Bedbenov V. S. Zorina A. A. Shumskaya M. (2018). Hydrogen peroxide participates in perception and transduction of cold stress signal in *Synechocystis*. *Plant Cell Physiol.* 59 1255–1264. 10.1093/pcp/pcy067 29590456

[B16] Galperin M. Y. (2006). Structural classification of bacterial response regulators: diversity of output domains and domain combinations. *J. Bacteriol.* 188 4169–4182. 10.1128/jb.01887-05 16740923 PMC1482966

[B17] Georg J. Voss B. Scholz I. Mitschke J. Wilde A. Hess W. R. (2009). Evidence for a major role of antisense RNAs in cyanobacterial gene regulation. *Mol. Syst. Biol.* 5:305. 10.1038/msb.2009.63 19756044 PMC2758717

[B18] Giner-Lamia J. Robles-Rengel R. Hernandez-Prieto M. A. Muro-Pastor M. I. Florencio F. J. Futschik M. E. (2017). Identification of the direct regulon of NtcA during early acclimation to nitrogen starvation in the cyanobacterium *Synechocystis* sp. PCC 6803. *Nucleic Acids Res.* 45 11800–11820. 10.1093/nar/gkx860 29036481 PMC5714215

[B19] He Q. Dolganov N. Bjorkman O. Grossman A. R. (2001). The high light-inducible polypeptides in Synechocystis PCC6803. Expression and function in high light. *J. Biol. Chem.* 276 306–314. 10.1074/jbc.m008686200 11024039

[B20] Horie Y. Ito Y. Ono M. Moriwaki N. Kato H. Hamakubo Y. (2007). Dark-induced mRNA instability involves RNase E/G-type endoribonuclease cleavage at the AU-box and SD sequences in cyanobacteria. *Mol. Genet. Genomics* 278 331–346. 10.1007/s00438-007-0254-9 17661085

[B21] Houot L. Floutier M. Marteyn B. Michaut M. Picciocchi A. Legrain P. (2007). Cadmium triggers an integrated reprogramming of the metabolism of *Synechocystis* PCC6803, under the control of the Slr1738 regulator. *BMC Genomics* 8:350. 10.1186/1471-2164-8-350 17910763 PMC2190772

[B22] Jiang H. B. Song W. Y. Cheng H. M. Qiu B. S. (2015). The hypothetical protein Ycf46 is involved in regulation of CO2 utilization in the cyanobacterium *Synechocystis* sp. PCC 6803. *Planta* 241 145–155. 10.1007/s00425-014-2169-0 25230699

[B23] Jiang X. Belasco J. G. (2004). Catalytic activation of multimeric RNase E and RNase G by 5′-monophosphorylated RNA. *Proc. Natl. Acad. Sci. U.S.A.* 101 9211–9216. 10.1073/pnas.0401382101 15197283 PMC438955

[B24] Jourdan S. S. McDowall K. J. (2008). Sensing of 5′ monophosphate by *Escherichia coli* RNase G can significantly enhance association with RNA and stimulate the decay of functional mRNA transcripts in vivo. *Mol. Microbiol.* 67 102–115. 10.1111/j.1365-2958.2007.06028.x 18078441

[B25] Kaberdin V. R. Miczak A. Jakobsen J. S. Lin-Chao S. Mcdowall K. J. Von Gabain A. (1998). The endoribonucleolytic N-terminal half of *Escherichia coli* RNase E is evolutionarily conserved in *Synechocystis* sp. and other bacteria but not the C-terminal half, which is sufficient for degradosome assembly. *Proc. Natl. Acad. Sci. U.S.A.* 95 11637–11642. 10.1073/pnas.95.20.11637 9751718 PMC21693

[B26] Kaneko T. Nakamura Y. Sasamoto S. Watanabe A. Kohara M. Matsumoto M. (2003). Structural analysis of four large plasmids harboring in a unicellular cyanobacterium, *Synechocystis* sp. PCC 6803. *DNA Res.* 10 221–228. 10.1093/dnares/10.5.221 14686584

[B27] Khemici V. Prados J. Linder P. Redder P. (2015). Decay-initiating endoribonucleolytic cleavage by RNase Y is kept under tight control via sequence preference and sub-cellular localisation. *PLoS Genet.* 11:e1005577. 10.1371/journal.pgen.1005577 26473962 PMC4608709

[B28] Kopf M. Klähn S. Scholz I. Matthiessen J. K. F. Hess W. R. Voß B. (2014). Comparative analysis of the primary transcriptome of *Synechocystis* sp. PCC 6803. *DNA Res.* 21 527–539. 10.1093/dnares/dsu018 24935866 PMC4195498

[B29] Kopfmann S. Hess W. R. (2013). Toxin-antitoxin systems on the large defense plasmid pSYSA of *Synechocystis* sp. PCC 6803. *J. Biol. Chem.* 288 7399–7409. 10.1074/jbc.m112.434100 23322786 PMC3591647

[B30] Laalami S. Bessieres P. Rocca A. Zig L. Nicolas P. Putzer H. (2013). *Bacillus subtilis* RNase Y activity *in vivo* analysed by tiling microarrays. *PLoS One* 8:e54062. 10.1371/journal.pone.0054062 23326572 PMC3542257

[B31] Laalami S. Putzer H. (2011). mRNA degradation and maturation in prokaryotes: the global players. *Biomol. Concepts* 2 491–506. 10.1515/bmc.2011.042 25962050

[B32] Laalami S. Zig L. Putzer H. (2014). Initiation of mRNA decay in bacteria. *Cell Mol. Life Sci.* 71 1799–1828. 10.1007/s00018-013-1472-4 24064983 PMC3997798

[B33] Lee K. Bernstein J. A. Cohen S. N. (2002). RNase G complementation of rne null mutation identifies functional interrelationships with RNase E in *Escherichia coli*. *Mol. Microbiol.* 43 1445–1456. 10.1046/j.1365-2958.2002.02848.x 11952897

[B34] Li H. Durbin R. (2009). Fast and accurate short read alignment with Burrows-Wheeler transform. *Bioinformatics* 25 1754–1760. 10.1093/bioinformatics/btp324 19451168 PMC2705234

[B35] Li de la Sierra-Gallay I. Zig L. Jamalli A. Putzer H. (2008). Structural insights into the dual activity of RNase. *J. Nat. Struct. Mol. Biol.* 15 206–212. 10.1038/nsmb.1376 18204464

[B36] Liponska A. Jamalli A. Kuras R. Suay L. Garbe E. Wollman F. A. (2018). Tracking the elusive 5′ exonuclease activity of Chlamydomonas reinhardtii RNase J. *Plant Mol. Biol.* 96 641–653. 10.1007/s11103-018-0720-2 29600502

[B37] Mackie G. A. (2013). RNase E: at the interface of bacterial RNA processing and decay. *Nat. Rev. Microbiol.* 11 45–57. 10.1038/nrmicro2930 23241849

[B38] Mäder U. Zig L. Kretschmer J. Homuth G. Putzer H. (2008). mRNA processing by RNases J1 and J2 affects *Bacillus subtilis* gene expression on a global scale. *Mol. Microbiol.* 70 183–196. 10.1111/j.1365-2958.2008.06400.x 18713320

[B39] Maeda S. Konishi M. Yanagisawa S. Omata T. (2014). Nitrite transport activity of a novel HPP family protein conserved in cyanobacteria and chloroplasts. *Plant Cell Physiol.* 55 1311–1324. 10.1093/pcp/pcu075 24904028

[B40] Mathy N. Benard L. Pellegrini O. Daou R. Wen T. Condon C. (2007). 5′-to-3′ exoribonuclease activity in bacteria: role of RNase J1 in rRNA Maturation and 5′ Stability of mRNA. *Cell* 129 681–692. 10.1016/j.cell.2007.02.051 17512403

[B41] Mathy N. Hebert A. Mervelet P. Benard L. Dorleans A. Li De La Sierra-Gallay I. (2010). Bacillus subtilis ribonucleases J1 and J2 form a complex with altered enzyme behaviour. *Mol. Microbiol.* 75 489–498. 10.1111/j.1365-2958.2009.07004.x 20025672

[B42] Michel K. P. Pistorius E. K. (2004). Adaptation of the photosynthetic electron transport chain in cyanobacteria to iron deficiency: the function of IdiA and IsiA. *Physiol. Plant* 120 36–50. 10.1111/j.0031-9317.2004.0229.x 15032875

[B43] Mohanty B. K. Kushner S. R. (2016). Regulation of mRNA decay in bacteria. *Annu. Rev. Microbiol.* 70 25–44. 10.1146/annurev-micro-091014-104515 27297126

[B44] Murashko O. N. Kaberdin V. R. Lin-Chao S. (2012). Membrane binding of *Escherichia coli* RNase E catalytic domain stabilizes protein structure and increases RNA substrate affinity. *Proc. Natl. Acad. Sci. U.S.A.* 109 7019–7024. 10.1073/pnas.1120181109 22509045 PMC3344982

[B45] Ohta H. Shibata Y. Haseyama Y. Yoshino Y. Suzuki T. Kagasawa T. (2005). Identification of genes expressed in response to acid stress in *Synechocystis* sp. PCC 6803 using DNA microarrays. *Photosynth. Res.* 84 225–230. 10.1007/s11120-004-7761-x 16049778

[B46] Pade N. Mikkat S. Hagemann M. (2017). Ethanol, glycogen and glucosylglycerol represent competing carbon pools in ethanol-producing cells of *Synechocystis* sp. PCC 6803 under high-salt conditions. *Microbiology* 163 300–307. 10.1099/mic.0.000433 28100303

[B47] Pei G. Sun T. Chen S. Chen L. Zhang W. (2017). Systematic and functional identification of small non-coding RNAs associated with exogenous biofuel stress in cyanobacterium *Synechocystis* sp. PCC 6803. *Biotechnol. Biofuels* 10:57. 10.1186/s13068-017-0743-y PMC5341163 28286552

[B48] Quinlan A. R. Hall I. M. (2010). BEDTools: a flexible suite of utilities for comparing genomic features. *Bioinformatics* 26 841–842. 10.1093/bioinformatics/btq033 20110278 PMC2832824

[B49] Redko Y. Condon C. (2010). Maturation of 23S rRNA in *Bacillus subtilis* in the absence of Mini-III. *J. Bacteriol.* 192 356–359. 10.1128/jb.01096-09 19880604 PMC2798235

[B50] Redko Y. Galtier E. Arnion H. Darfeuille F. Sismeiro O. Coppee J. Y. (2016). RNase J depletion leads to massive changes in mRNA abundance in *Helicobacter pylori*. *RNA Biol.* 13 243–253. 10.1080/15476286.2015.1132141 26726773 PMC4829309

[B51] Robinson J. T. Thorvaldsdottir H. Winckler W. Guttman M. Lander E. S. Getz G. (2011). Integrative genomics viewer. *Nat. Biotechnol.* 29 24–26. 10.1038/nbt.1754 21221095 PMC3346182

[B52] Robinson M. D. Mccarthy D. J. Smyth G. K. (2010). edgeR: a Bioconductor package for differential expression analysis of digital gene expression data. *Bioinformatics* 26 139–140. 10.1093/bioinformatics/btp616 19910308 PMC2796818

[B53] Rott R. Zipor G. Portnoy V. Liveanu V. Schuster G. (2003). RNA polyadenylation and degradation in cyanobacteria are similar to the chloroplast but different from *Escherichia coli*. *J. Biol. Chem.* 278 15771–15777. 10.1074/jbc.m211571200 12601000

[B54] Schein A. Sheffy-Levin S. Glaser F. Schuster G. (2008). The RNase E/G-type endoribonuclease of higher plants is located in the chloroplast and cleaves RNA similarly to the *E. coli* enzyme. *RNA* 14 1057–1068. 10.1261/rna.907608 18441049 PMC2390796

[B55] Scholz I. Lange S. J. Hein S. Hess W. R. Backofen R. (2013). CRISPR-Cas systems in the cyanobacterium *Synechocystis* sp. PCC6803 exhibit distinct processing pathways involving at least two Cas6 and a Cmr2 protein. *PLoS One* 8:e56470. 10.1371/journal.pone.0056470 23441196 PMC3575380

[B56] Schuck A. Diwa A. Belasco J. G. (2009). RNase E autoregulates its synthesis in *Escherichia coli* by binding directly to a stem-loop in the rne 5′ untranslated region. *Mol. Microbiol.* 72 470–478. 10.1111/j.1365-2958.2009.06662.x 19320830 PMC2857391

[B57] Shahbabian K. Jamalli A. Zig L. Putzer H. (2009). RNase Y, a novel endoribonuclease, initiates riboswitch turnover in Bacillus subtilis. *EMBO J.* 28 3523–3533. 10.1038/emboj.2009.283 19779461 PMC2782095

[B58] Sharwood R. E. Halpert M. Luro S. Schuster G. Stern D. B. (2011). Chloroplast RNase J compensates for inefficient transcription termination by removal of antisense RNA. *RNA* 17 2165–2176. 10.1261/rna.028043.111 22033332 PMC3222129

[B59] Stern D. B. Goldschmidt-Clermont M. Hanson M. R. (2010). Chloroplast RNA metabolism. *Annu. Rev. Plant Biol.* 61 125–155. 20192740 10.1146/annurev-arplant-042809-112242

[B60] Strahl H. Turlan C. Khalid S. Bond P. J. Kebalo J. M. Peyron P. (2015). Membrane recognition and dynamics of the RNA degradosome. *PLoS Genet.* 11:e1004961. 10.1371/journal.pgen.1004961 25647427 PMC4372235

[B61] Summerfield T. C. Sherman L. A. (2008). Global transcriptional response of the alkali-tolerant cyanobacterium *Synechocystis* sp. strain PCC 6803 to a pH 10 environment. *Appl. Environ. Microbiol.* 74 5276–5284. 10.1128/aem.00883-08 18606800 PMC2546634

[B62] Wang J. Chen L. Huang S. Liu J. Ren X. Tian X. (2012). RNA-seq based identification and mutant validation of gene targets related to ethanol resistance in cyanobacterial *Synechocystis* sp. PCC 6803. *Biotechnol. Biofuels* 5:89. 10.1186/1754-6834-5-89 23259593 PMC3564720

[B63] Yajnik V. Godson G. N. (1993). Selective decay of *Escherichia coli* dnaG messenger RNA is initiated by RNase E. *J. Biol. Chem.* 268 13253–13260. 7685758

[B64] Yeremenko N. Jeanjean R. Prommeenate P. Krasikov V. Nixon P. J. Vermaas W. F. (2005). Open reading frame ssr2016 is required for antimycin A-sensitive photosystem I-driven cyclic electron flow in the cyanobacterium *Synechocystis* sp. PCC 6803. *Plant Cell Physiol.* 46 1433–1436. 15946981 10.1093/pcp/pci147

[B65] Yeremenko N. Kouril R. Ihalainen J. A. D’haene S. Van Oosterwijk N. Andrizhiyevskaya E. G. (2004). Supramolecular organization and dual function of the IsiA chlorophyll-binding protein in cyanobacteria. *Biochemistry* 43 10308–10313. 15301529 10.1021/bi048772l

[B66] Yu N. Y. Wagner J. R. Laird M. R. Melli G. Rey S. Lo R. (2010). PSORTb 3.0: improved protein subcellular localization prediction with refined localization subcategories and predictive capabilities for all prokaryotes. *Bioinformatics* 26 1608–1615. 20472543 10.1093/bioinformatics/btq249 PMC2887053

[B67] Zerulla K. Ludt K. Soppa J. (2016). The ploidy level of *Synechocystis* sp. PCC 6803 is highly variable and is influenced by growth phase and by chemical and physical external parameters. *Microbiology* 162 730–739. 26919857 10.1099/mic.0.000264

[B68] Zhang J. Y. Deng X. M. Li F. P. Wang L. Huang Q. Y. Zhang C. C. (2014). RNase E forms a complex with polynucleotide phosphorylase in cyanobacteria via a cyanobacterial-specific nonapeptide in the noncatalytic region. *RNA* 20 568–579. 24563514 10.1261/rna.043513.113 PMC3964918

