# Supplementary material for: Impact of RNase E and RNase J on Global mRNA Metabolism in the Cyanobacterium Synechocystis PCC6803
Source: Front Microbiol. 2020 Jun 3;11:1055. doi: 10.3389/fmicb.2020.01055 (PMC7283877; doi:10.3389/fmicb.2020.01055)
Supplement: FIGURE S1 — Primary and secondary structure of Sy RNase E and Sy RNase J annotated using the PROMALS3D tool. (A) Multiple protein sequence alignment of RNase E/G proteins from Synechocystis and E. coli using the structure template of Ec RNase E (PDB ID: 2bx2, chain L). Conserved domains are underlined: RNase H in green, S1 in blue, 5′sensor in pink and DNase I in red; the C1 to C4 motifs are highlighted in cyano. α-helix predictions are colored in red and β-strand in blue. (B) Multiple protein sequences alignment of Sy RNase J, Bs RNase J1/J2 and Cr RNase J using the structure template of Tt RNaseJ1 (PDB ID: 3BK1 chain (A). The β-lactamase motifs are in yellow, the β-CASP motifs in green. The β-CASP domain is underlined in blue and the C-terminal domain in pink. In (A) and (B) the consensus secondary structure (Consensus_ss) is indicated by h (α-helix) and e (β-strand). In the consensus amino acid sequence (Consensus_aa) conserved amino acids are in bold and uppercase letters; aliphatic: l; aromatic: @; hydrophobic: h; alcohol: o; polar residues: p; tiny: t; small: s; bulky residues: b; positively charged: +; negatively charged: −; charged: c. [file Data_Sheet_1.pdf]

A

```
SyRNaseE                1   -------MPKQIVIAE--KHQVAAVFWKDQIQELVVSTGS--QQVGDIYLGLVDNILPSIDAAFINIGDT   59
EcRNaseE                1   -------MKRMLINATQQEELRVALVDGQRLYDLDIESPGHEQKKANIYKGKITRIEPSLEAAFVDYGA-   62
EcRNaseG                1   -------MTAELLVNVTPSETRVAYIDGGILQEIHIEREARRGIVGNIYKGRVSRVLPGMQAAFVDIGL-   62
2bx2.pdb_chainL_s001    1   AHIEGRHMKRMLINATQQEELRVALVDGQRLYDLDIESPGHEQKKANIYKGKITRIEPSLEAAFVDYGA-   69
Consensus_aa:               .......M..bll.s...pc..hAhh....lb-l.lpp.t...b.tsIYbG.lsplbPthpAAFlshG..
Consensus_ss:                         eeeee    eeeeeeee  eeeeeee           eeeeeeee      eeeeee
                                                    RNaseH                        S1

SyRNaseE               60   EKNGFIHVSDLGPVRLRR--------TAGSISELLSPQQRVLVQVMKEPTGNKGPRLTGNISMPGRYMVL  121
EcRNaseE               63   ERHGFLPLKEIAREYFPANYS---AHGRPNIKDVLREGQEVIVQIDKEERGNKGAALTTFISLAGSYLVL  129
EcRNaseG               63   DKAAFLHASDIMPHTECVAGEEQKQFTVRDISELVRQGQDLMVQVVKDPLGTKGARLTTDITLPSRYLVF  132
2bx2.pdb_chainL_s001   70   ERHGFLPLKEIAREYFP----------RPNIKDVLREGQEVIVQIDKEERGNKGAALTTFISLAGSYLVL  129
Consensus_aa:               -+.tFl.hp-l....b.............sIp-llp..QclhVQl.K-..GsKGs.LTs.IohstpYhVh
Consensus_ss:                  eeeehhhhhhhh              hhhh     eeeeeeee        eeeeeeee   eeee
                                                                   5′sensor

SyRNaseE              122   MPYGRG-VNLSRRINREEERSRLRALAVLIKPPGMGLLVRTEAEDVPEDAIIEDLENLQKQWELVQQQAM  190
EcRNaseE              130   MPNNPRAGGISRRIEGDDRTELKEALASLELPEGMGLIVRTAGVGKSAEALQWDLSFRLKHWEAIKKAAE  199
EcRNaseG              133   MPGASH-VGVSQRIESESERERLKKVVAEYCDEQGGFIIRTAAEGVGEAELASDAAYLKRVWTKVMERKK  201
2bx2.pdb_chainL_s001  130   MPNNPRAGGISRRI------ELKEALASLELPEGMGLIVRTAGVGKSAEALQWDLSFRLKHWEAIKKAAE  193
Consensus_aa:               MP.s...sslSpRI......p.bc.lhsbb.s...GhllRT.t.s.s...l..Dh...b+.Wp.lbp..b
Consensus_ss:                        eeee   hhhhhhhhhhhhhh    eeeee          hhhhhhhhhhhhhhhhhhhhhhh

SyRNaseE              191   TRSAPMLLDRDDDFIKRVLRDMYSSEVNRIVVDTPAGMKRIKQQLMNWDQGRLPEGVLIDCHRESLSILE  260
EcRNaseE              200   SRPAPFLIHQESNVIVRAFRDYLRQDIGEILIDNPKVLELARQHIAALGRP--DFSSKIKLYTGEIPLFS  267
EcRNaseG              202   RPQTRYQLYGELALAQRVLRDFADAELDRIRVDSRLTYEALLEFTSEYIPE---MTSKLEHYTGRQPIFD  268
2bx2.pdb_chainL_s001  194   SRPAPFLIHQESNVIVRAFRDYLRQDIGEILIDNPKVLELARQHIAALGRP--DFSSKIKLYTGEIPLFS  261
Consensus_aa:               p..h.hbl..-.shh.RhhRDhhp.-lscI.lDs..shc.h.p.h..h......bssblch@p.pbslhp
Consensus_ss:                    eeee    hhhhhhhhh      eeee  hhhhhhhhhhhhhh        eee      hhh
                                                                  RNaseH

SyRNaseE              261   YFRVNATIREALKPRVDLPSGGYIIIEPTEALTVIDVNSGSFTHSANSRETVLWTNYEAATEIARQLKLR  330
EcRNaseE              268   HYQIESQIESAFQREVRLPSGGSIVIDSTEALTAIDINSARATRGGDIEETAFNTNLEAADEIARQLRLR  337
EcRNaseG              269   LFDVENEIQRALERKVELKSGGYLIIDQTEAMTTVDINTGAFVGHRNLDDTIFNTNIEATQAIARQLRLR  338
2bx2.pdb_chainL_s001  262   HYQIESQIESAFQREVRLPSGGSIVIDSTEALTAIDINSAR----GDIEETAFNTNLEAADEIARQLRLR  327
Consensus_aa:               h@plpspIppAhp.cVcL.SGG.llI-.TEAhThlDlNot......s.c-Thh.TNhEAhp.IARQL+LR
Consensus_ss:               h  hhhhhhhhh  eee     eeeee    eeeeee          hhhhhhhhhhhhhhhhhhhhhh
                                                            DNaseI
```

```
SyRNaseE            331  NIGGVIIIDFIDMDSHKDQLQLLEHFNRCLETDKARPQIAQLTELGLVELTRKRQGQNLYELFGQPCPEC  400
EcRNaseE            338  DLGGLIVIDFIDMTPVRHQRAVENRLREAVRQDRARIQISHISRFGLLEMSRQRLSPSLGESSHHVCPRC  407
EcRNaseG            339  NLGGIIIIDFIDMNNEDHRRRVLHSLEQALSKDRVKTSVNGFSALGLVEMTRKRTRESIEHVLCNECPTC  408
2bx2.pdb_chainL_s001 328  DLGGLIVIDFIDMTPVRHQRAVENRLREAVRQDRARIQISHISRFGLLEMSRQRLSPSLGESSHHVCPRC  397
Consensus_aa:            slGGlIlIDFIDMss.ccp..lbpphpptlppD+h+.pls.ho.hGLlEhoRpR...sl.c...p.CPpC
Consensus_ss:                eeeee      hhhhhhhhhhhhhhhh     eee       eeeeee      hhhhh

SyRNaseE            401  GGLGHLVELPGEKGFVSLSPTAVNSSIPPRLVEKPILSPPVAKVNDLP---------KKEEAKISSPLDL  461
EcRNaseE            408  SGTGTVRDNESLSLSILRLIEEEALKENTQEVHAIVPVPIASYLLNEKRSAVNAIETRQDGVRCVIVPND  477
EcRNaseG            409  HGRGTVKTVETVCYEIMREIVRVHHAYDSDRFLVYASPAVAEALKGEESHSLAEVE-IFVGKQVKVQIEP  477
2bx2.pdb_chainL_s001 398  SGTGTVRDNESLSLSILRLIEEEALKENTQEVHAIVPVPIASYLLNEKRSAVNAIETRQDGVRCVIVPND  467
Consensus_aa:            .G.Ghl.p..s....l..........bssp.h...h.ss.h..l.sb..........b...ph....p.
Consensus_ss:               eeeeehhhhhhhhhhhhhhhhh     eeeeee hhhhhhhh   hhhhhhhh hh   eeeeee

                         Zn Link
SyRNaseE            462  LF-HPNYQEQGDRD--------------------------------------------------------  474
EcRNaseE            478  QMETPHYHVLRVRKGEETPTLSYMLPKLHEEAMALPSEEEFAERKRPEQPALATFAMPDVPPAPTPAEPA  547
EcRNaseG            478  LYNQEQFDVVMM----------------------------------------------------------  489
2bx2.pdb_chainL_s001 468  QMETPHYHVLRVRKGEETPTLSYMLPKLHEEAM-------------------------------------  500
Consensus_aa:            bh.p.p@p..............................................................
Consensus_ss:                  eeeee
                            C1

SyRNaseE            475  ----------------------------------------------------------SNRR--------  478
EcRNaseE            548  APVVAPAPKAAPATPAAPAQPGLLSRFFGALKALFSGGEETKPTEQPAPKAEAKPERQQDRRKPRQNNRR  617
EcRNaseG                 ----------------------------------------------------------------------
2bx2.pdb_chainL_s001     ----------------------------------------------------------------------
Consensus_aa:            ......................................................................
Consensus_ss:                                                                      hhhh

SyRNaseE            479  ----------------------RRRRRGSEFSEKENIKSVGISRSKGPSPSPTKEKVTGTAPPRRERPSR  526
EcRNaseE            618  DRNERRDTRSERTEGSDNREENRRNRRQAQQQTAETRESRQQAE-----VTEKARTADEQQAPRRERSRR  682
EcRNaseG                 ----------------------------------------------------------------------
2bx2.pdb_chainL_s001     ----------------------------------------------------------------------
Consensus_aa:            ......................................................................
Consensus_ss:                                  hhhhhh
                                                   C2

SyRNaseE            527  RVEKTLVPVDVAMTTLEQDIYARMGISPLIKTEYADQDPRSFMVSVVTAGAALEGNTNGSGSLVNAVITT  596
EcRNaseE            683  RNDDKRQAQQEAKALNVE--------EQSVQETEQEER--------VRPVQPRRKQRQLNQKVRYEQSVA  736
EcRNaseG                 ----------------------------------------------------------------------
2bx2.pdb_chainL_s001     ----------------------------------------------------------------------
Consensus_aa:            ......................................................................
Consensus_ss:

                                                 C3
```

```
SyRNaseE                597  VDNGDNGDNVPSDGLTIVSEVTAPTPVIEQPREETVEPEQVVLPQLDDETPAAPVAEESAPIETKKRP--  664
EcRNaseE                737  EEAVVAPVVEETVAA--EPIVQEAPAPRTELVKVPLPVVAQTAPEQQEENNADNRDNGGMPRRSRRSPRH  804
EcRNaseG                     ----------------------------------------------------------------------
2bx2.pdb_chainL_s001         ----------------------------------------------------------------------
Consensus_aa:                ......................................................................
Consensus_ss:

SyRNaseE                665  ----GRRRRS-------------------SAE--------------------------------------  674
EcRNaseE                805  LRVSGQRRRRYRDERYPTQSPMPLTVACASPELASGKVWIRYPIVRPQDVQVEEQREQEEVHVQPMVTEV  874
EcRNaseG                     ----------------------------------------------------------------------
2bx2.pdb_chainL_s001         ----------------------------------------------------------------------
Consensus_aa:                ......................................................................
Consensus_ss:                      hhh
                                    C4

SyRNaseE                     ----------------------------------------------------------------------
EcRNaseE                875  PVAAAIEPVVSAPVVEEVAGVVEAPVQVAEPQPEVVETTHPEVIAAAVTEQPQVITESDVAVAQEVAEQA  944
EcRNaseG                     ----------------------------------------------------------------------
2bx2.pdb_chainL_s001         ----------------------------------------------------------------------
Consensus_aa:                ......................................................................
Consensus_ss:

SyRNaseE                     ----------------------------------------------------------------------
EcRNaseE                945  EPVVEPQEETADIEEVVETAEVVVAEPEVVAQPAAPVVAEVAAEVETVAAVEPEVTVEHNHATAPMTRAP 1014
EcRNaseG                     ----------------------------------------------------------------------
2bx2.pdb_chainL_s001         ----------------------------------------------------------------------
Consensus_aa:                ......................................................................
Consensus_ss:

SyRNaseE                     -----------------------------------------------
EcRNaseE               1015  APEYVPEAPRHSDWQRPTFAFEGKGAAGGHTATHHASAAPARPQPVE 1061
EcRNaseG                     -----------------------------------------------
2bx2.pdb_chainL_s001         -----------------------------------------------
Consensus_aa:                ...............................................
Consensus_ss:
```

B

```
SyRNaseJ                         ----------------------------------------------------------------------
BsRNaseJ1                        ----------------------------------------------------------------------
BsRNaseJ2                        ----------------------------------------------------------------------
CrRNaseJ                      1  MQGGQVLAGVSPASTSPAPAAQRARSLSTRNVVAQADNSERWRTQGGRGGGGGRGGGRGGGRGGGGGGN   70
rnj_3BK1.pdb_chainA_s001         ----------------------------------------------------------------------
Consensus_aa:                    ......................................................................
Consensus_ss:
```

```
SyRNaseJ                  1   -----------MAK----------------------------NTQTQALKILPLGGLHEIGKNTCVFEYD   31
BsRNaseJ1                 1   -----------MK-----------------------------FVKNDQTAVFALGGLGEIGKNTYAVQFQ   30
BsRNaseJ2                 1   -----------MK-----------------------------KKNTENVRIIALGGVGEIGKNLYVIEID   30
CrRNaseJ                 71   RRGKTYVSESGMTLKSYVASDGQEHLGSALQKRRYKPFPGPPTNGGPPLRILPIGGLGEIGMNCMLAGVG  140
rnj_3BK1.pdb_chainA_s001  1   ------------------------------------------GPQDHVEIIPLGGMGEIGKNITVFRFR   27
Consensus_aa:                 ...........M.............................s.s..lcIlslGGlGEIGbNhhlh.h.
Consensus_ss:                                                            eeeeeee          eeeeeee

SyRNaseJ                 32   DEILLLDAGLAFPTDDMHGVNVVLPDMTYLRENREKIKGMVVTHGHEDHIGGIAYHLKQFDI----PIIY   97
BsRNaseJ1                31   DEIVLIDAGIKFPEDELLGIDYVIPDYTYLVKNEDKIKGLFITHGHEDHIGGIPYLLRQVN-----IPVY   95
BsRNaseJ2                31   SDIFVVDAGLMHPENEMLGIDVVIPDISYLIERADRVKAIFLTHGHDENIGGVFYLLNKLS-----VPVY   95
CrRNaseJ                141   DRYVVIDAGLMFPDFSDLGMQKILPDTDFLAQWKDRIEALIITHGHEDHIGALPWVVPALD---PATPIY  207
rnj_3BK1.pdb_chainA_s001 28   DEIFVLDGGLAFPEEGMPGVDLLIPRVDYLIEHRHKIKAWVLTHGHEDHIGGLPFLLPMIFGKESPVPIY   97
Consensus_aa:                 DchhllDAGLhFP-.p.hGhp.llPDhs@Lhp.c-+IcthhlTHGHEDHIGtls@ll..ls.....hPlY
Consensus_ss:                  eeeeee             hhhhhhhhhhhhh       eeeee    hhhh   hhhhhhh       eee
                                    M1                                         M2

SyRNaseJ                 98   GPRLAMALLRDKLEEAGMLE-RTNLQTVSPREMVRLGKSFVVEFIRNTHSIADSYCLAIHTPLGVVMHSG  166
BsRNaseJ1                96   GGKLAIGLLRNKLEEHGLLR-QTKLNIIGEDDIVKFR-KTAVSFFRTTHSIPDSYGIVVKTPPGNIVHTG  163
BsRNaseJ2                96   GTKLTLALLREKLKQYGHNR-KTDLREIHSKSVITFE-STKVSFFRTIHSIPDSVGVSFKTSLGSIVCTG  163
CrRNaseJ                208   ASSFVMQLVKRRLTEYNLWD-EKRFITFDMRQRFQAG-PFEIEPVRVTHSIPDCCGLIMRSDEGTIVHTG  275
rnj_3BK1.pdb_chainA_s001 98   GARLTLGLLRGKLEEFGLRPGAFNLKEISPDDRIQVGRYFTLDLFRMTHSIPDNSGVVIRTPIGTIVHTG  167
Consensus_aa:                 tsphhh.Ll+p+LpE@sh.c.ppph.phs.cp.hph...h.lp.hRhTHSIPDthGlhh+osbGsIVHTG
Consensus_ss:                   hhhhhhhhhhhhh             eeee     eeee    eeeeeee       eeeeeeee    eeeeee
                                                                                  M3

SyRNaseJ                167   DFKIDHTPIDGEFFDLQKVAEYGEKGVLCLLSDSTNAEVPGITPSEASVIPNLDRVFSQA--EGRLMVTT  234
BsRNaseJ1               164   DFKFDFTPV-GEPANLTKMAEIGKEGVLCLLSDSTNSENPEFTMSERRVGESIHDIFRKV--DGRIIFAT  230
BsRNaseJ2               164   DFKFDQTPALNQTCDIGEIAKIGNSGVLALLSDSANAERPGYTPSEAAVSGEISDALYNS--QNRVIIAV  231
CrRNaseJ                276   DWKIDENPLDGEQFDRELFMALGSEPVALMMSDSTNVLSPGRTLSEQVVHDSLVEKVLEHNGRGRVICTQ  345
rnj_3BK1.pdb_chainA_s001 168   DFKLDPTPIDGKVSHLAKVAQAGAEGVLLIADATNAERPGYTPSEMEIAKELDRVIGRA--PGRVFVTT  235
Consensus_aa:                 D@KhD.sPl.Gc.hc..bhh.hGpcsVhhhhSDSTNhbpPG.T.SE..V..plsc.h.ph..pGRlhhhp
Consensus_ss:                               hhhhhhh        eeee                hhhhhhhhhhhhhh      eeeee
                              M4                                A
                                                                                  β-CASP

SyRNaseJ                235   FASSVHRVNIILSLAQKHQRKVAVVGRSMLNVIAHARKLGYIKCPDNLFVPLKAARNLPDQQQLILTTGS  304
BsRNaseJ1               231   FASNIHRLQQVIEAAVQNGRKVAVFGRSMESAIEIGQTLGYINCPKNTFIEHNEINRMPANKVTILCTGS  300
BsRNaseJ2               232   FASNINRIQQVIHAAAQNGRKIAVAGKNLQSVLQLARKLGYIEADDELFISVQDVKKYPKREVAIITAGS  301
CrRNaseJ                346   FASNLHRLYGVKRAADAAGRKICFVGASLNHYLEAAWRDGRAPFDPKELMPTEQLRHANPNEVLIVTTGS  415
rnj_3BK1.pdb_chainA_s001 236   FASHIHRIQSVIWAAEKYGRKVAMEGRSMLKFSRIALELGYLKVKDR-LYTLEEVKDLPDHQVLILATGS  304
Consensus_aa:                 FASslHRlb.VbpAA...GRKlthhG.Sh.phlphA.c.G.h.hssp.hhshppl+chssppVhIlhTGS
Consensus_ss:                    hhhhhhhhhhhhh    eeeee  hhhhhhhhhhhh         hhh    hhhhh        eeeeee
```

```
SyRNaseJ                  305  QGEPLAAMTRISNGEHPQIKIRQGDTVVFSANPIPGNTIAVVNTIDRLMMQGANVIYGKHQGIHVSGHAS  374
BsRNaseJ1                 301  QGEPMAALSRIANGTHRQISINPGDTVVFSSSPIPGNTISVSRTINQLYRAGAEVIHGPLNDIHTSGHGG  370
BsRNaseJ2                 302  QGEPLAALTRMANKAHKQLNIEEGDTVVIASTPIPGQELIYSKTVDLLARAGAQVIFAQK-RVHVSGHGS  370
CrRNaseJ                  416  QGEPRAQLSMAARDQSNILKTLPGDLLLYSAKVIPGNEGKVTKMLNALAGQGARVRQSRADNLHTSGHAY  485
rnj_3BK1.pdb_chainA_s001  305  QGQPMSVLHRLAFEGHAKMAIKPGDTVILSSSPIPGNEEAVNRVINRLYALGAYVLYPPTYKVHASGHAS  374
Consensus_aa:                  QGEP.A.Lo.hAp..p.blph..GDhllhStpsIPGNp..Vs+hls.Lh..GApV.bt.h.plHhSGHt.
Consensus_ss:                     hhhhhhhh       eee    eeeee      hhhhhhhhhhhhhh   eeeee   eeeee
                                                                                                  B
SyRNaseJ                  375  QEEHKMLLALTRPKFFVPVHGEHRMLVKHSQMAQAQGIPSENIVIVNNGDVIELTGDRIRVAGQVPSGIE  444
BsRNaseJ1                 371  QEEQKLMLRLIKPKFFMPIHGEYRMQKMHVKLATDCGIPEENCFIMDNGEVLALKGDEASVAGKIPSGSV  440
BsRNaseJ2                 371  QEELKLMINLLKPKYLIPVNGEYRMQKAHSKIAEETGMKRSDIFLIEKGDVVEFRGQNVKIGDKVPYGNI  440
CrRNaseJ                  486  QEELVELLQSVRPQHFLPVHGEYAFLTEHALLAKTR-AGVNFTDVIRNGEMLAVRERR--NRNTVSTGSM  552
rnj_3BK1.pdb_chainA_s001  375  QEELKLILNLTTPRFFLPWHGEVRHQMNFKWLAESMSRPPEKTLIGENGAVYRLTRETFEKVGEVPHGVL  444
Consensus_aa:                  QEEh.bhLp.h+Pp@FlPlHGE@.hb..HsblAps.ths.p.h.lhcNG-hl.lp.cphp..spVspGsh
Consensus_ss:                  hhhhhhhhhh    eeeee   hhhhhhhhhhhhh    hhheee    eeeee    eeeeeeee   e
                                                  C

SyRNaseJ                  445  LVDQAGIVH----------------------------ESTMAERQQMAEDGLVTVAA---ALSK-TGTLL  482
BsRNaseJ1                 441  YIDGSGIGDIG--------------------------NIVLRDRRILSEEGLVIVVV---SIDMDDFKIS  481
BsRNaseJ2                 441  LIDGLGVGDIG--------------------------NIVLRDRRLLSQDGILIVVI---TLDKQKKHLV  481
CrRNaseJ                  553  AVAAARGGFEAPTMVKYDGEEPTYFFNDGGKGTGTRTEMEIDMRGTMAMEGVVVVGVDVIRRSAGDYGLG  622
rnj_3BK1.pdb_chainA_s001  445  YVDGLGVGDIT--------------------------EEILADRRHMAEEGLVVITA---LAGEDP----  481
Consensus_aa:                  hlsth..G.bs..........................pb.l..R.hhtb-GlVlVsh.....s..s..l.
Consensus_ss:                  eee                                  hhhhhhhhhhhh  eeeeee   eee    ee

SyRNaseJ                  483  AYPEVHCRGVVMTIQPKLLEELIVRTIENFLTERWSEFTHGSNGSTEVSWNALQKELESSLQRLIKRELQ  552
BsRNaseJ1                 482  AGPDLISRGFVYMRESGDLINDAQELISNHLQKVMERK--------TTQWSEIKNEITDTLAPFLYEKTK  543
BsRNaseJ2                 482  SGPEIITRGFVYVRESEGLIVQATELVRSIVTEATETS--------NVEWSTLKQAMRDALNQFLYEKTK  543
CrRNaseJ                  623  CGVRVTTRGMWTDE--GKLPAELTAAVENAVARLSGT----------CSLVEVERAAIDAVKRRCIAFNN  680
rnj_3BK1.pdb_chainA_s001  482  -VVEVVSRGFVK--AGERLLGEVRRMALEALKNGVRE---------KKPLERIRDDIYYPVKKFLKKATG  539
Consensus_aa:                  tssclhoRGhhh...s..L.sphp.hlpshl.c...p.........phphsplcp.h.sslpp.hb.bsp
Consensus_ss:                     eeeee  eee   hhhhhhhhhhhhhhhhhhhh             hhhhhhhhhhhhhhhhhhhh
                                                                C-terminal domain

SyRNaseJ                  553  SSPMVLLMLQTDTPIELDQVPQNV--------STVSA---------------------------------  581
BsRNaseJ1                 544  RRPMILPIIMEV----------------------------------------------------------  555
BsRNaseJ2                 544  RKPMIIPIIMEV----------------------------------------------------------  555
CrRNaseJ                  681  KRPEVIAIAYEHDPRDAHVAEVAEARADAAARANRARGEPEDAGGPASVLTPRRKAQNPWESGAAGSSIR  750
rnj_3BK1.pdb_chainA_s001  540  RDPMILPVVIEG----------------------------------------------------------  551
Consensus_aa:                  +cPbllslhhE...........................................................
Consensus_ss:                     eeeeeeee
```

```
SyRNaseJ                582  ----------------TSATPAPRKKVVLTK---------TPEPKVKAKPEKKVVTTAEPSAQ-PVSTTK  625
BsRNaseJ1                    ----------------------------------------------------------------------
BsRNaseJ2                    ----------------------------------------------------------------------
CrRNaseJ                751  PSAPSTSATAPVRSAPASPAASSRAPGVLVPGGARRTRLPAPEPAPEPAPE-------EPPAPAPAPAAR  813
rnj_3BK1.pdb_chainA_s001     ----------------------------------------------------------------------
Consensus_aa:                ......................................................................
Consensus_ss:

SyRNaseJ                626  VYRRSRKRSTT----------SVSS---------------------------------------------  640
BsRNaseJ1                    ----------------------------------------------------------------------
BsRNaseJ2                    ----------------------------------------------------------------------
CrRNaseJ                814  KVRRSR--TTTAPAPDSDGIISISGSTDDDEDAAAAAGPASGSVAADEDSKLSRMLRARKAAASTRRTRA  881
rnj_3BK1.pdb_chainA_s001     ----------------------------------------------------------------------
Consensus_aa:                ......................................................................
Consensus_ss:

SyRNaseJ                     ---------------------------------------
BsRNaseJ1                    ---------------------------------------
BsRNaseJ2                    ---------------------------------------
CrRNaseJ                882  DKGEEGAGTLTPLDPKVIADRRQRNPRDRPHSIEDPDYG  920
rnj_3BK1.pdb_chainA_s001     ---------------------------------------
Consensus_aa:                .......................................
Consensus_ss:
```

Supplementary Figure 1. **Primary and secondary structure of *Sy* RNase E and *Sy* RNase J (PROMALS3D)**. A) Multiple protein sequence alignment of RNase E/G proteins from *Synechocystis* and *E. coli* using the structure template of *Ec* RNase E (PDB ID: 2bx2, chain L). Conserved domains are underlined: RNase H in green, S1 in blue, 5'sensor in pink and DNase I in red; the C1 to C4 motifs are highlighted in cyano. α-helix predictions are colored in red and β-strand in blue. B) Multiple protein sequences alignment of *Sy* RNase J, *Bs* RNase J1/J2 and *Cr* RNase J using the structure template of *Tt* RNaseJ1 (PDB ID: 3BK1 chain A). The β-lactamase motifs are in yellow, the β-CASP motifs in green. The β-CASP domain is underlined in blue and the C-terminal domain in pink. In A and B the consensus secondary structure (Consensus_ss) is indicated by h (α-helix) and e (β-strand). In the consensus amino acid sequence (Consensus_aa) conserved amino acids are in bold and uppercase letters; aliphatic: l; aromatic: @; hydrophobic: h; alcohol: o; polar residues: p; tiny: t; small: s; bulky residues: b; positively charged: +; negatively charged: -; charged: c.

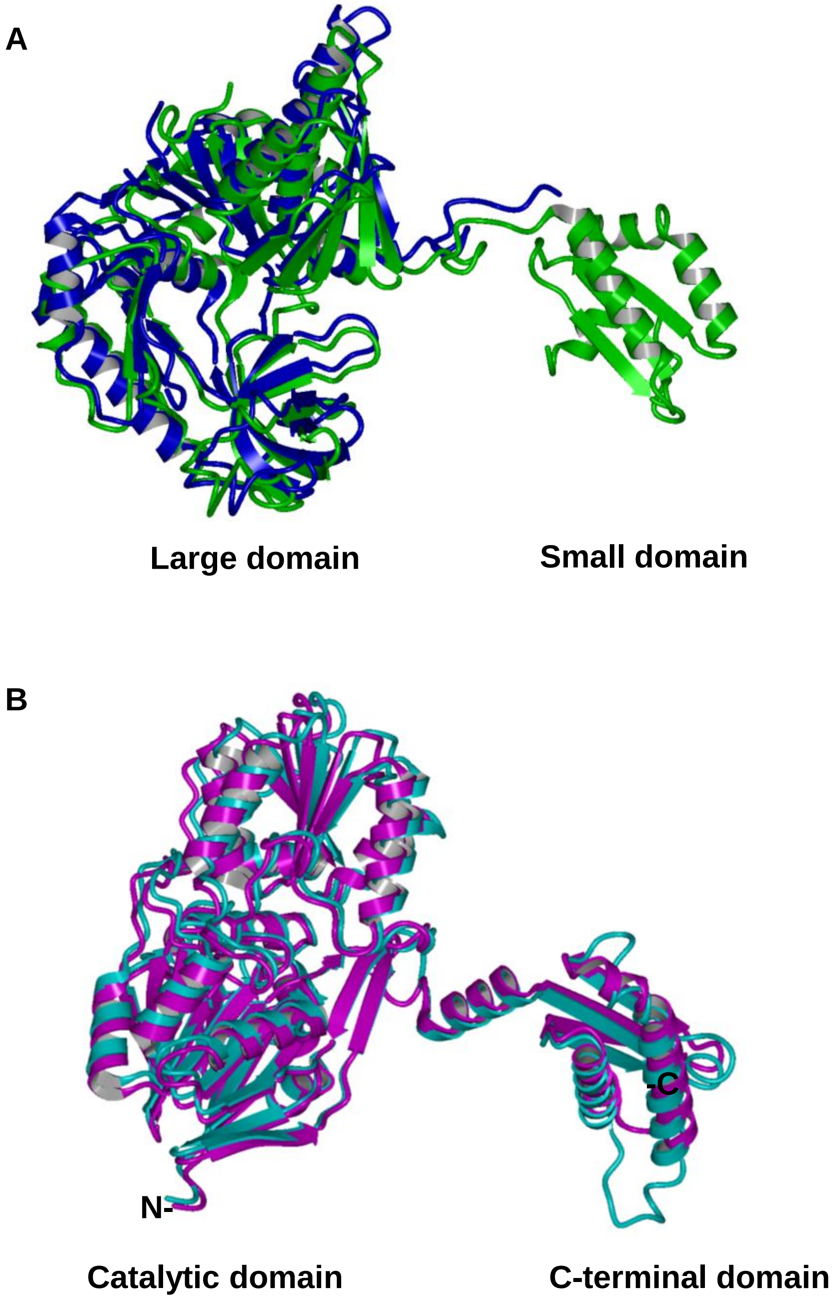

Supplementary Figure 2. Homology-based model of the *Sy* RNase and *Sy* RNase J structures. (A) The modeled catalytic region of *Sy* RNase E (blue) superimposed on the *E. coli* crystal structure PDB 2bx2, chain L (green). (B) The modeled structure of *Sy* RNase J (cyano) superimposed on the *T. thermophilus* crystal structure PDB 3BK1 (magenta).

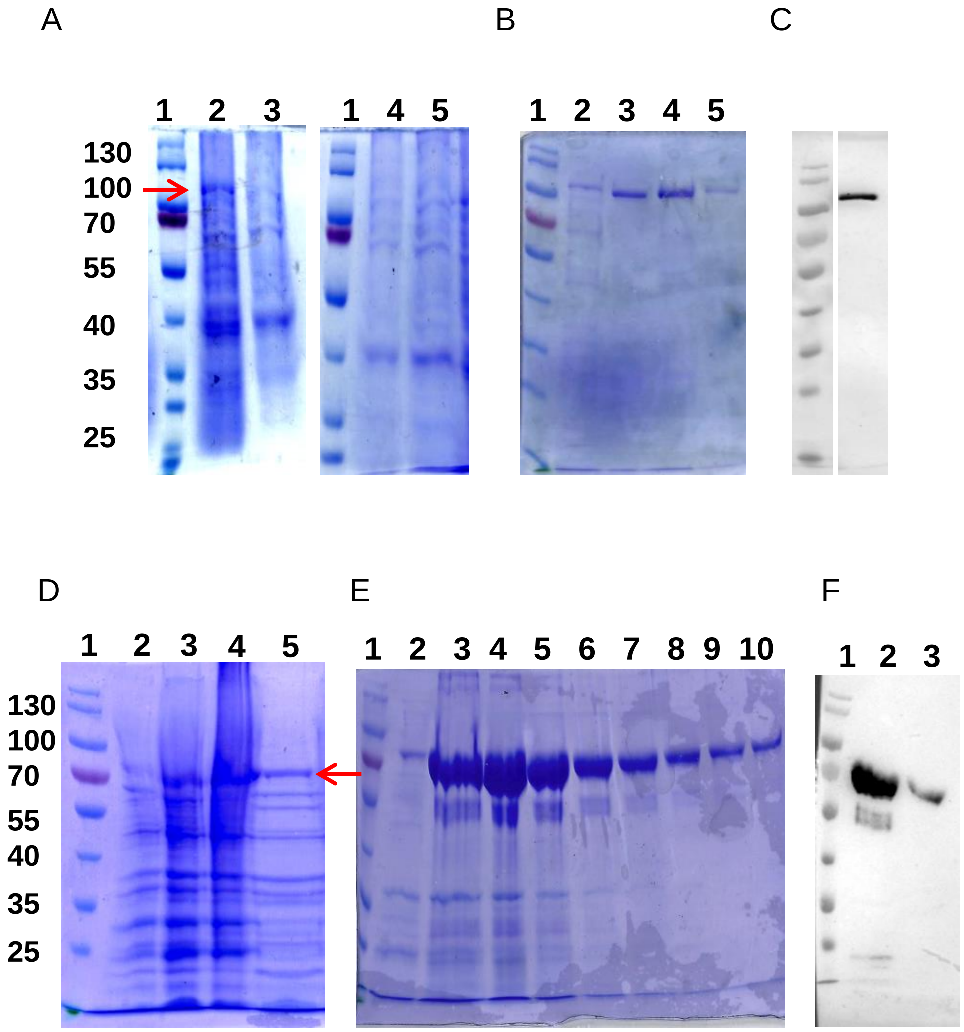

Supplementary Figure 3. **Expression and purification of recombinant *Sy* RNase E (A-C) and *Sy* RNAse J (D-F) in *E. coli.*** SDS-PAGE was carried out on a 10% polyacrylamide gel and proteins were detected by Coomassie Brilliant Blue R-250 stain. A) Induction of Sy RNase E: Lane 1, MW standards. Lanes 2 and 3, insoluble fractions after and before induction with 1 mM IPTG, respectively. Lanes 4 and 5, soluble fraction after and before induction, respectively. B) Purification of *Sy* Rnase J His-tagged recombinant protein. Lane 1, MW standards. Lanes 2 to 5, purified RNase E. C) Immunoblot with anti-His tag antibody on collected pooled dialyzed proteins from lanes 3-5 of the gel in B). D) Induction of *Sy* RNase J. Lane 1, MW standards; Lanes 2 and 3, whole cell lysates before and after induction, respectively. Lanes 4 and 5, insoluble fractions and soluble fraction after induction. E) Purification of *Sy* Rnase J His-tagged recombinant protein. Lane 1, MW standards. Lanes 2 to 8, purified RNase J. F) Immunoblot with anti-His tag antibody. Pooled purified dialyzed proteins from lanes 1 to 4 (Lane 1) and from lanes 5 to 9, (Lane 2), respectively.

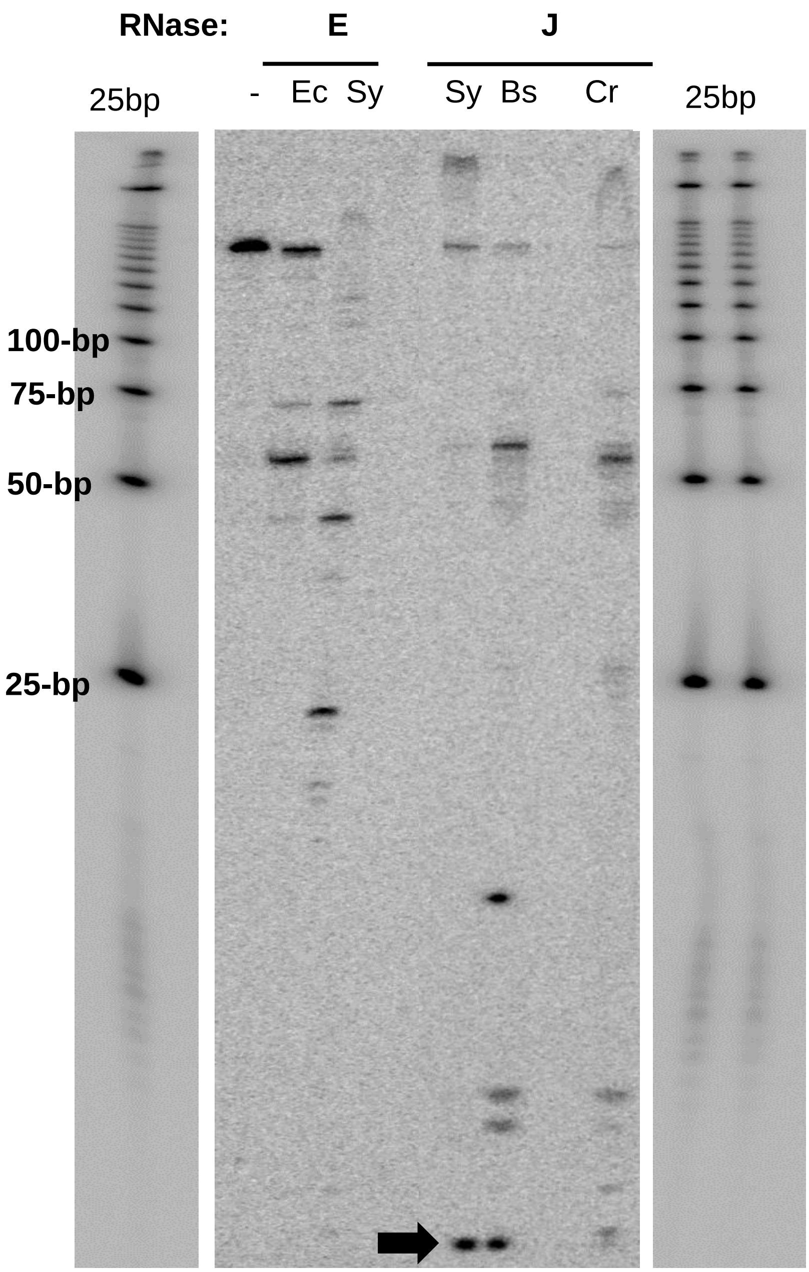

Supplementary Figure 4. *In vitro* activity of *Sy* RNase E and *Sy* RNase J. A 5'-monophosphorilated (P) - 32P end-labeled *yitJ* was incubated with RNase E (E) from *E. coli* (*Ec*) and *Synechocystis* (*Sy*) and RNase J (J) from *Synechocystis* (*Sy*), *B. subtilis* (*Bs*), and *C.reinhardtii* (*Cr*). As control (lane 1) the transcript was incubated with the reaction buffer only (-). The reactions were separated on a 20% urea PAGE gel. The mononucleotides corresponding to liberated 5'-terminal nucleotide are at the bottom of the gel (depicted by an arrow) and are indicative of a 5'-3' exoribonuclease activity. L= 32P-labeled 25 bp DNA ladder.

A

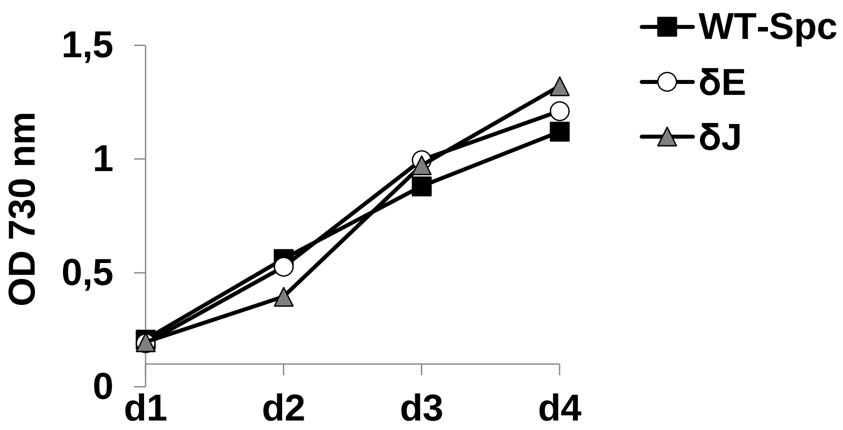

B

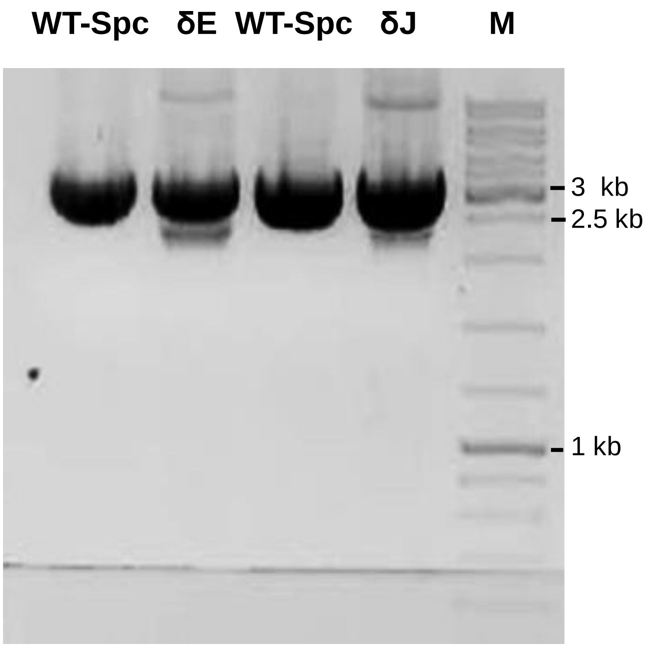

Supplementary Figure 5. Genetic instability of rne and rnj deletion. A) Growth curves of *Synechocystis* mutants and WT strains in the absence of antibiotic in BG11 medium. B) PCR analysis of wild-type (WT-Spc) and mutant strains (δE and δJ) grown in the absence of spectinomycin. The almost exclusive presence of the WT gene copy (~3.5 kb band) over the mutant gene copy (~2.5 kb and ~2.4 kb) in the mutant strains highlights the rapid loss of the partial gene deletions in the absence of antibiotic pressure.

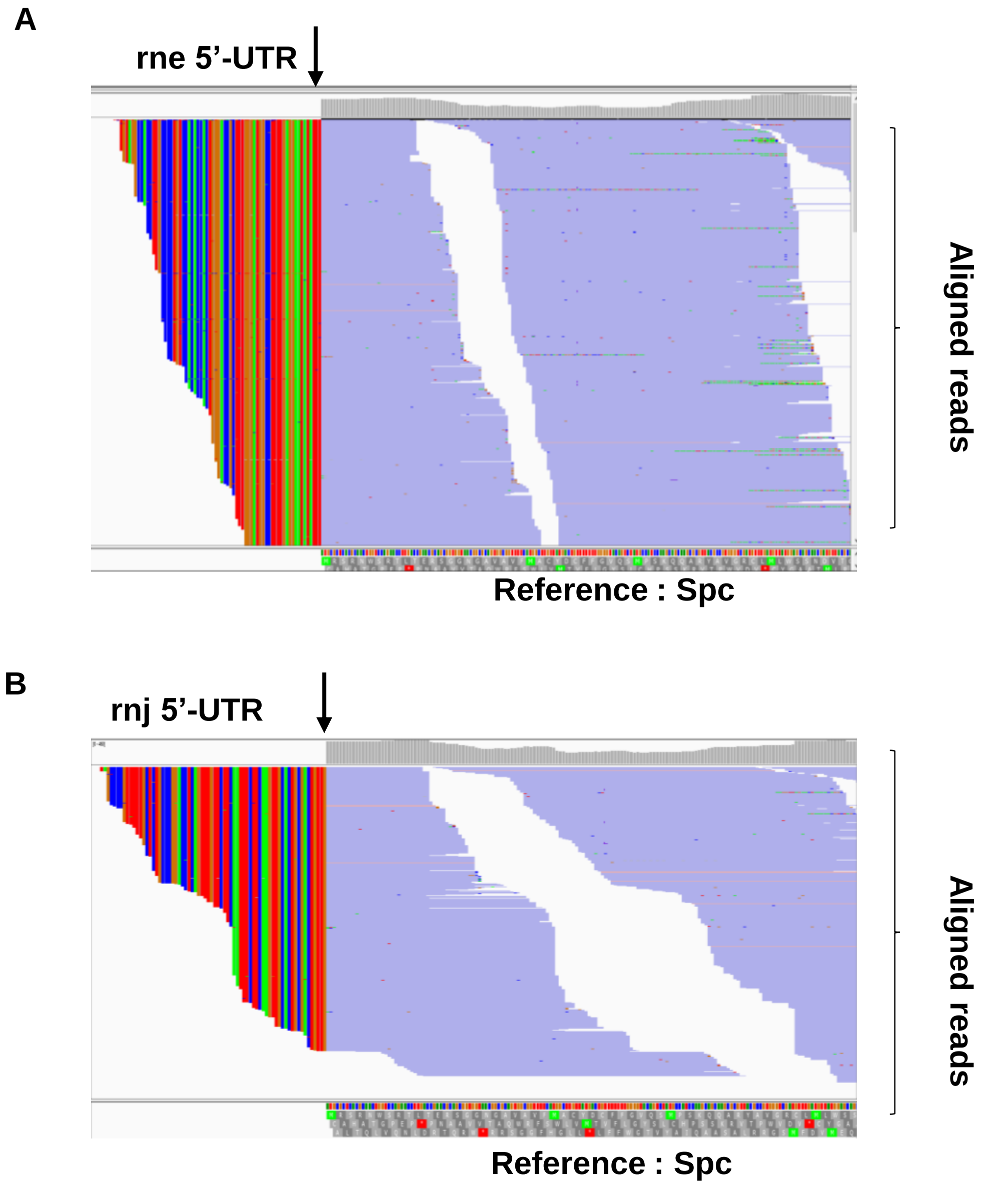

Supplementary Figure 6. Browser view of RNA-Seq soft-clipped reads showing the integration of the Spc cassette at the intended locus. Reads from replicates S4 (A) and S7 (B) are shown as representative examples. In these snapshots, the first 160-nt of Spc sequence is shown at the bottom. The soft-clipped portion at the 5' end of the reads that do not match to the Spc coding sequence are shown as multicolour "rainbow" (C: blue, U: red; A: green; G: brown). The breakpoint of soft-clipped reads from reference is indicated by the arrow. The soft-clipped sequence corresponds to the sequence of 5'-UTR of *Sy* rne (A) and *Sy* rnj (B).

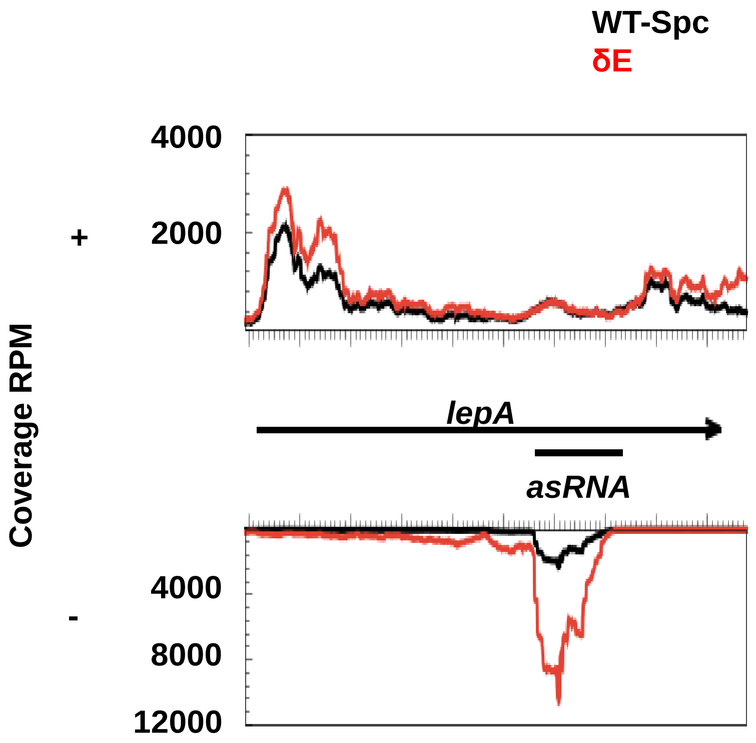

Supplementary Figure 7. Antisense coverage over lepA. RNA-Seq coverage over the *lepA* gene in the WT (black) and in the δE mutant (red), on the sense (+) strand (on the top) and on the antisense (-) strand (on the bottom). Arrow represents the *lepA* ORF and its orientation on the genome; the line represents the asRNA identified by (Georg et al., 2009). RPM: reads per million
